# Innovations in Pain Management for Abdominoplasty Patients: A Systematic Review

**DOI:** 10.3390/jpm14111078

**Published:** 2024-10-26

**Authors:** Bryan Lim, Ishith Seth, Jevan Cevik, Jeevan Avinassh Ratnagandhi, Gabriella Bulloch, Paola Pentangelo, Alessandra Ceccaroni, Carmine Alfano, Warren M. Rozen, Roberto Cuomo

**Affiliations:** 1 Department of Plastic and Reconstructive Surgery, Frankston Hospital, Peninsula Health, Frankston, VIC 3199, Australia; ishith.seth@monash.edu (I.S.); jratnagandhi@phcn.vic.gov.au (J.A.R.); warren.rozen@monash.edu (W.M.R.); 2 Faculty of Medicine and Surgery, Monash University, Melbourne, VIC 3800, Australia; 3 Faculty of Medicine and Surgery, The University of Melbourne, Melbourne, VIC 3010, Australia; gbulloch@student.unimelb.edu.au; 4 Department of Medicine and Surgery, University of Salerno, 84084 Salerno, Italy; ppentangelo@unisa.it (P.P.); aceccaroni@unisa.it (A.C.); calfano@unisa.it (C.A.); 5 Plastic Surgery Unit, Department of Medicine, Surgery and Neuroscience, University of Siena, 53100 Siena, Italy; roberto.cuomo2@unisi.it

**Keywords:** abdominoplasty, analgesia, opioid, blocks, pain

## Abstract

**Background/Objectives**: Abdominoplasties are prevalent surgical procedures for improving lower abdominal contours, necessitating effective pain management. Insufficient management can increase opioid usage, dependency risks, and adverse effects. This review investigates various strategies in abdominoplasty pain management, aiming to reduce opioid dependence and improve patient care. **Methods**: A comprehensive systematic literature search (MEDLINE, Cochrane, PubMed, Web of Science, EMBASE) was conducted, spanning from their inception to January 2024, using keywords such as ‘abdominoplasty’ and ‘postoperative pain management’. Included studies focused on nonopioid interventions in adults, encompassing various study designs. Non-English publications and those not meeting outcome criteria were excluded. Bias in studies was assessed using specific tools for randomized and non-randomized trials. **Results**: Thirty-five studies, published between 2005 and 2024, were included, involving 3636 patients with an average age of 41.8. Key findings highlighted the effectiveness of transversus abdominis plane blocks in reducing opioid use and pain. Pain pump catheters also showed promise in improving pain management and reducing opioid dependency. Local anesthetics demonstrated varying degrees of efficacy, while other alternatives like ketamine and NSAIDs successfully reduced postoperative pain and opioid requirements. The bias assessment of the RCTs revealed “low” and “some concerns” ratings, indicating a need for more detailed methodology reporting and management of missing data. The cohort studies generally attained “moderate” risks of bias, primarily due to confounding variables and outcome data reporting. **Conclusions**: Nonopioid analgesics show potential in postoperative pain management for abdominoplasties, but further research is needed to confirm their effectiveness and optimize patient care.

## 1. Introduction

Abdominoplasty, also known as a tummy tuck, is a widely performed surgical procedure that seeks to restore the normal anatomical structure of the lower abdominal skin and address abdominal contour deformities. This is achieved by removing excess skin and fat from the middle and lower abdomen and tightening the abdominal wall muscles and fascia to achieve a favorable cosmetic result [1]. The procedure ranked as the fourth most common surgical procedure in 2018 with over 100,000 performed cases according to the International Society of Aesthetic Plastic Surgery (ISAPS) [2].

A critical aspect of postoperative care in abdominoplasty is pain management, as when prolonged or severe, it becomes a significant concern for patients and can lead to increased opioid consumption [3,4]. Surgical patients are nearly four times more likely to receive post-discharge opioids than nonsurgical patients, conferring a heightened risk of long-term opioid use. Historically, morphine and other opioids have been the cornerstone of managing postoperative pain, despite their well-known side effects and risk of addiction [5]. It is estimated that those prescribed opioids within seven days of surgery have a 44% increased likelihood of becoming long-term opioid users within a year [6,7].

Poor management of postoperative pain raises other concerns, increases the risk of chronic pain, and decreases quality of life. Conversely, excessive opioid use can result in adverse effects like nausea, dizziness, and most critically, respiratory depression, and potential opioid dependency. There is also evidence suggesting that excessive opioid consumption may induce hyperalgesia, a heightened sensitivity to pain [7]. Therefore, it is essential to utilize a multimodal approach to analgesia when managing patients post abdominoplasty to effectively manage postoperative discomfort and avoid long-term side effects.

Alternative methods to manage postoperative pain are diverse but require higher levels of ongoing care compared with oral analgesics. These include the use of an epidural catheter block and transversus abdominis plane block (TAP-B) [8]. Although effective, the latter is unsuitable for many patients, while the former requires an anesthesiologist and ultrasound guidance. Liposomal bupivacaine may also be considered although its availability varies globally [9]. Finally, infusion pain pumps with local wound catheters are a novel strategy that may be used for several days and are suitable for ambulatory use [10].

This systematic review investigates the latest advancements in pain management techniques specific to abdominoplasty and examines the efficacy of various methods including the use of pain pump catheters (PPCs), multimodal analgesia strategies, and the role of patient-controlled analgesia (PCA) in reducing postoperative opioid consumption [5]. By evaluating the current state of research and clinical applications, this systematic review aims to provide a nuanced understanding of the evolving landscape of pain management in abdominoplasty, ultimately guiding practitioners toward more effective and patient-oriented approaches.

## 2. Materials and Methods

### 2.1. Literature Search

**Figure 1:**
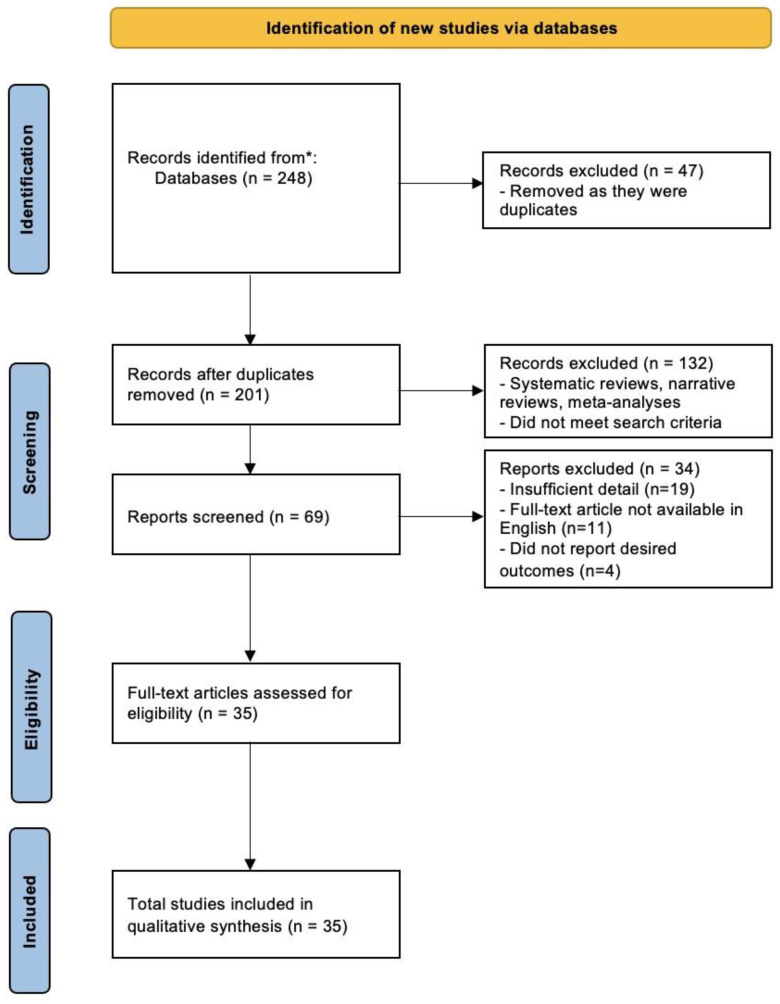
PRISMA flow diagram detailing the search strategy.

Two independent authors (BL and IS) performed the search, focusing on exploring a broad spectrum of evidence-based postoperative pain relief methods following abdominoplasty. The databases searched included MEDLINE, Cochrane, PubMed, Web of Science, and EMBASE from their inception to August 2024. Artificial intelligence tools, namely Elicit and Perplexity, were employed to aid in the search process and also to assess for eligibility. The search was guided by a range of keywords and terms specifically chosen to capture the breadth of the topic: ‘abdominoplasty’, ‘lipoabdominoplasty’, ‘panniculectomy’, ‘circumferential lipectomy’, ‘mini abdominoplasty’, ‘truncal dermatolipectomy’, ‘postoperative pain management’, ‘postoperative analgesia’, ‘pain control’, ‘analgesia’, and ‘pain’. Aligning with the narrative review approach, literature was explored without a specific time frame, allowing for a comprehensive understanding of the evolution and current state of pain management in abdominoplasty (Figure 1). The review follows the PRISMA guidelines and was registered prospectively on the International Prospective Register of Systematic Reviews. The 2020 PRISMA checklist and PRISMA abstract checklist were completed.

### 2.2. Study Selection

The selection criteria were structured according to the Participants, Intervention, Comparison, Outcomes and Study design (PICOS) format. The purpose was to evaluate pain management strategies following abdominoplasty. The intervention focused on nonopioid analgesic options, including nonsteroidal anti-inflammatory drugs, cyclo-oxygenase-2 (COX-2) inhibitors, acetaminophen, nefopam, metamizole, corticosteroids, and alpha-2 agonists. The control groups included either opioid-based pain management or placebo interventions. Outcomes of interest were primarily pain assessment scores, and secondarily side effects or complications related to the interventions. Studies included participants aged 18 and older, and accepted study types were randomized controlled trials (RCTs), prospective or retrospective cohort/comparative studies, case-control studies, and case series. Studies were excluded if they were not published in English and did not report the desired outcomes, with primary outcomes focusing on pain assessment scores and secondary outcomes on side effects. Meta-analyses, systematic reviews, economic analyses, animal studies, cadaver studies, other narrative reviews, editorials, and studies focusing primarily on opioid analgesics were also excluded. Following the search, duplicates were removed using endnote software version 20, and two independent authors (BL and IS) screened abstracts for relevance. A third author (GB) was consulted when disagreements arose. Subsequently, full texts were screened to ensure compliance with the inclusion criteria.

### 2.3. Data Extraction

Data relevant to postoperative pain management in abdominoplasty were extracted and summarized into tables using an Excel spreadsheet. Study details including title, authors, publication year, country, study design as well as demographic data (sample size, mean age/age range), types of interventions used for postoperative pain control, surgical outcomes measured, the timing of interventions (preoperative, intraoperative, postoperative), specifics of the surgical procedures, dosages and methods of analgesic administration, any complications noted in treatment versus placebo groups, and patient satisfaction levels were extracted (Table 1 and Table 2). The title, abstract, and full text of the studies were also screened to ensure their eligibility for inclusion (Figure 1).

### 2.4. Bias Assessment

**Figure 2:**
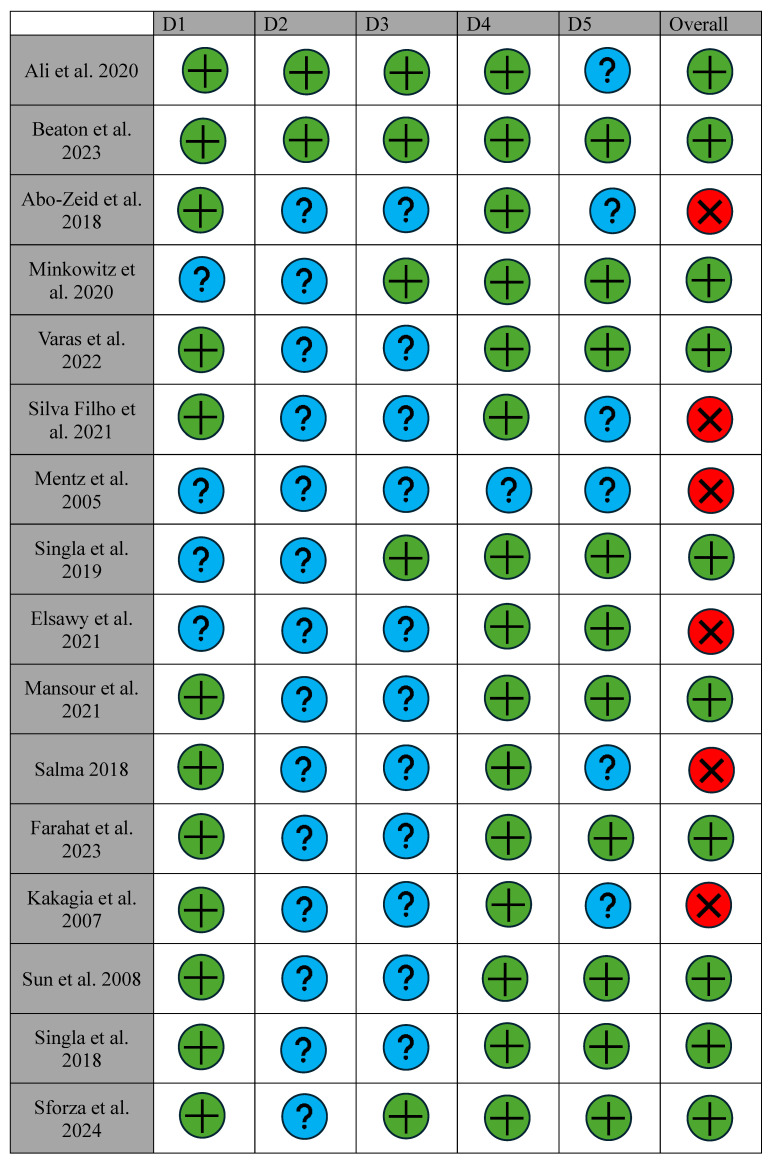

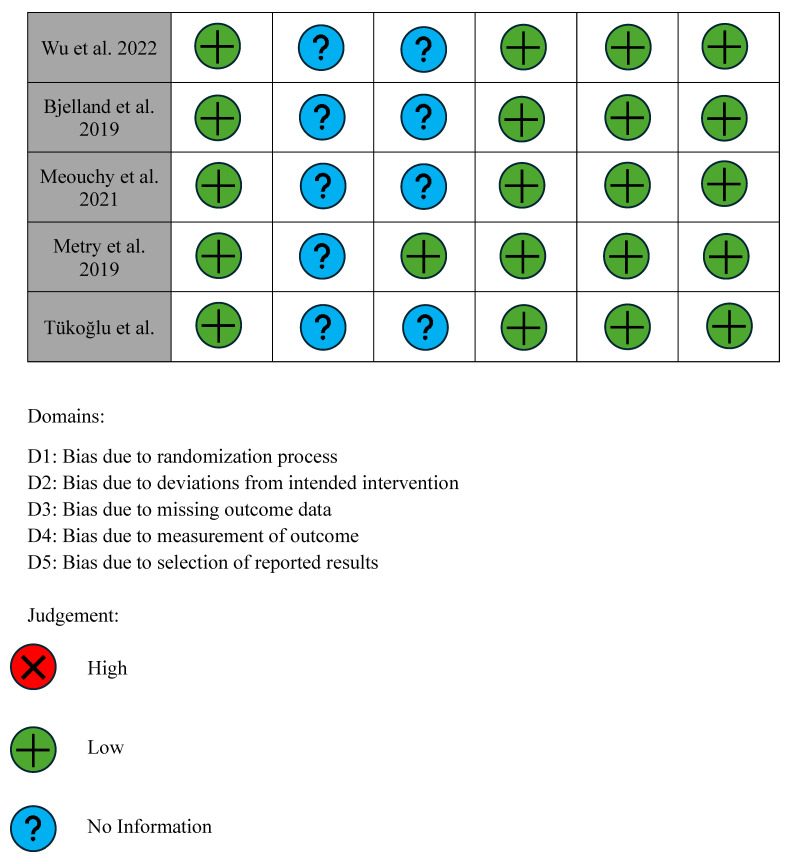
Risk of bias assessment of shortlisted studies [6,17,20,21,22,23,24,26,27,29,32,33,34,35,36,37,38,39,40,41,43].

**Figure 3:**
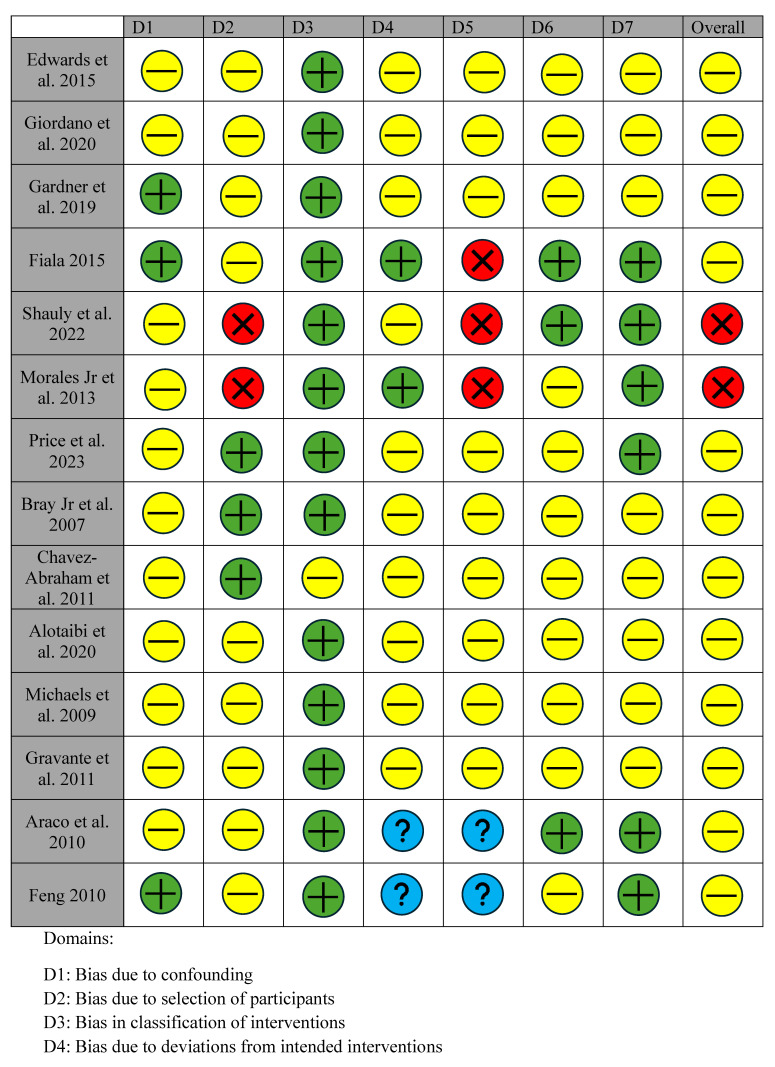

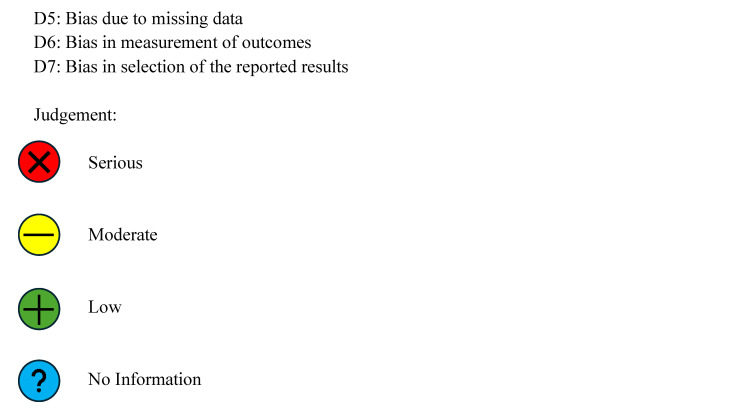
ROBINS-I assessment of shortlisted studies [3,7,10,14,15,16,18,19,25,28,30,31,44,45].

Given the narrative approach of this review, biases were qualitatively identified by reviewing the methodologies of the included studies, especially in the context of their design, data collection, and analysis. All RCTs were evaluated for their risk of bias utilizing the Cochrane risk of bias (ROB 2 tool) tool for randomized trials (Table 3, Figure 2) [42,43]. The randomization process, missing outcome data, measurement of outcome, selection of reported results, and overall risk of bias were all analyzed and recorded by one reviewer. Each bias was rated as either “low”, “some concerns”, or “high”. The cohort studies, both prospective and retrospective, were analyzed using the risk of bias in non-randomized studies of interventions (ROBINS-I) 2016 tool (Table 4, Figure 3) [44]. This tool assesses seven domains of possible bias including confounding, participant selection, intervention classification, deviation from expected interventions, missing data, outcome measurement, and selection of reported results. Each domain was categorized as having “low”, “moderate”, “serious” or “critical” risk of bias. For the purposes of this review, “serious” or “critical” risks were designated as having a “high” risk of bias.

### 2.5. Comparing Studies with Patients Who Underwent Rectus Plication and Diastasis Correction vs. Those Without

Considering the varying levels of pain associated with different abdominoplasty techniques is crucial for both patients and healthcare providers. Abdominoplasty combined with rectus muscle plication and diastasis correction typically induces stronger pain compared to pure cutaneous/subcutaneous procedures. Pain intensity in abdominoplasty is closely tied to skin, muscle, and fascia tension, meaning that comparing pain levels across different techniques requires assessing the comparability of skin tension. Therefore, in this study, we will systematically compare pain management strategies, delineating between studies that incorporated rectus plication and/or diastasis correction and those that did not.

### 2.6. GRADE Assessment of All Included Studies

The authors applied the Grading of Recommendations, Assessment, Development, and Evaluation (GRADE) framework to systematically assess the quality of all included studies. This approach will involve evaluating key domains, including risk of bias, inconsistency, indirectness, imprecision, and publication bias, for each outcome of interest. The evidence will then be rated as high, moderate, low, or very low quality, providing a robust and transparent evaluation of the overall strength of the findings.

## 3. Results

### 3.1. Literature Findings

The initial search produced 248 results. After removing 47 duplicates, an additional 132 were excluded for being systematic or narrative reviews, meta-analyses, or not meeting the search criteria. This left 69 studies, of which 19 were further eliminated due to insufficient detail. Eleven studies were excluded because their full texts were not in English, and 4 more were discarded for not reporting the desired outcomes. There were 35 studies published between 2005 and 2023 which were included (Figure 1).

This comprised 4 prospective cohort studies, 10 retrospective studies, and 21 RCTs. Table 1 lists the characteristics of the 11 included studies where participants underwent rectus plication. Table 2 details the 24 studies that do not explicitly mention performing rectus plication.

### 3.2. Study Characteristics

A total of 3636 patients were included. The range of ages of all patients across all included studies was 18–75. The age range of participants was not reported in 20 of the included studies, while 10 studies did not include the average ages. All studies had divided their patients into one, two, or three study groups. Studies by Edwards et al., Shauly et al., and Morales Jr. et al., investigated only one group of patients with their respective analgesic interventions [13,16,22]. Eighteen studies investigated one control against one therapeutic group, while the remaining 6 studied two intervention groups against one control group.

### 3.3. Risk of Bias Assessments

Among the 21 RCTs, 15 were assessed as having an overall “low” bias rating, whereas the remaining 6 were rated as having “high” bias (Table 3). In the case of the 14 cohort studies, 12 received “low” overall bias ratings, with the remaining 2 classified as “high” (Table 4).

### 3.4. Studies Investigating Nerve Blocks in Patients Who Underwent Rectus Plication

Abo-Zeid et al.’s research compared outcomes between TAP-Bs, rectus sheath blocks (RS-Bs), and subcutaneous infiltrations (Sci). They revealed that TAP-Bs surpass RS-Bs and Sci in extending analgesia duration and diminishing the need for morphine [12]. This finding suggests that TAP-Bs may offer superior anesthesia for these patients.

Gardner et al. and Fiala’s investigations concur with these findings, demonstrating that TAP-Bs administered before abdominoplasty notably reduce narcotic usage, pain scores, and delay the initial request for pain medication [2,8,14,15]. Their studies underscore TAP-Bs’ role in facilitating early ambulation compared to standard nerve blocks or local anesthetics, affirming their efficacy as a postoperative analgesic in abdominoplasty procedures.

Bjelland’s study found that the QL-B, when used as part of a multimodal pain regimen for post-bariatric patients undergoing standard full abdominoplasty, did not significantly reduce opioid requirements, postoperative pain, postoperative nausea and vomiting, or other side effects [19]. Technical difficulties were encountered in 26% of subjects during block administration, but successful block placement failure was low. Despite the meticulous design, including triple blinding to eliminate bias, no significant benefits of QL-B were observed, contrasting with previous studies that suggested an opioid-sparing effect. The study suggests a potential minimal benefit within the first 12 h postoperatively, but this was deemed clinically insignificant, questioning the addition of QL-B to multimodal pain regimens for such procedures [19].

### 3.5. Studies Investigating Nerve Blocks in Patients Without Rectus Plication

Salama et al.’s studies corroborate the efficacy of ultrasound-guided TAP-Bs in delivering effective pain relief after abdominoplasty, advocating for their utilization [29]. Collectively, these studies contribute significantly to the body of knowledge on the effectiveness of various nerve blocks, particularly TAP-Bs, in enhancing postoperative pain management.

Wu et al. studied 180 patients undergoing laparoscopic cholecystectomy, finding no significant differences in postoperative pain, sleep quality, or mobility between groups receiving a local anesthetic infiltration (LAI) alone, LAI with an ultrasound-guided TAP-B, or LAI with TAP-B and RS-B [39]. However, the LAI group reported higher satisfaction with pain management within 48 h post-surgery [39]. They concluded that while ultrasound-guided nerve blocks effectively manage pain, LAI alone may be preferable for patient satisfaction and ease of use in clinical practice, as they eliminate skin suture tension pains.

In a study by Sforza et al., a randomized, controlled, double-blind trial with 30 female patients demonstrated that the S-PECS block, a combination of serratus anterior and PECS-2 blocks, significantly reduced pain following breast augmentation surgery compared to saline injection. Patients in the S-PECS group were also 74% less likely to need additional pain medication [37]. The findings suggest that the S-PECS block is an effective technique for managing postoperative pain in breast surgery with potential for wider application [37].

Meouchy’s study demonstrated the effectiveness of the quadratus lumborum block (QL-B) in abdominoplasty, highlighting its benefits in reducing postoperative pain and opioid consumption. Patients in the ropivacaine group, who received this block, required less morphine and tramadol postoperatively, and reported lower pain levels on the numeric rating scale [36]. Additionally, the quality of recovery was significantly better in the ropivacaine group, underscoring the potential of this technique to enhance post-surgical recovery [36].

Feng’s study revealed significant benefits of using a combination of intercostal, ilioinguinal, iliohypogastric, and pararectus blocks for abdominoplasty patients, marking the first report of successful long-term pain relief associated with this procedure [3]. The treatment group experienced significantly less pain, required fewer narcotics, and spent less time in the recovery room compared to the control group. Additionally, these patients reported lower pain scores at home, had less nausea, consumed less pain medication, and resumed normal activities more quickly. This pain-block technique reduced recovery time and facilitated a faster return to work and daily activities [3].

Araco’s study on aesthetic abdominal surgery with the TAP-B, performed without ultrasound guidance, highlighted its safety and effectiveness in reducing postoperative opioid analgesia requirements. The study, involving seventy-five patients, recorded no intra- or postoperative complications, demonstrating the procedure’s safety. Patients receiving the TAP block showed a significant reduction in the need for analgesia during the first 12 postoperative hours. However, it was noted that patients with a higher BMI and those with a larger flap resected were more likely to experience anesthetic block failure, necessitating additional postoperative analgesia [38].

In their study, Farahat et al. evaluated the analgesic efficacy of ultrasound-guided ESP-B versus ultrasound-guided TAP-B in a multimodal analgesic strategy for abdominoplasty patients [31]. The trial included 69 females with ASA I and II physical status, aged 25 to 65 years, who underwent general anesthesia. Participants were divided into three groups: a control group receiving only standard general anesthesia; a TAP-B group receiving the block in addition to general anesthesia; and an ESP-B group also receiving the block alongside general anesthesia. Results showed that both ESP-B and TAP-B significantly reduced postoperative opioid consumption and delayed the first request for analgesics compared to the control group. ESP-B provided longer-lasting analgesia and reduced postoperative heart rate, while TAP-B was simpler and quicker to perform. The study concluded that both techniques effectively enhance postoperative analgesia in abdominoplasty patients.

Elsawy et al. compared the effectiveness of ultrasound-guided ESP-B and TAP-B for postoperative pain management in abdominoplasty [27]. In this randomized trial of 51 patients, the ESP-B group (*n* = 25) showed significantly lower visual analog scale (VAS) scores at 8 and 12 h, a longer time to request the first analgesic dose (9.16 ± 1.07 h vs. 7.65 ± 0.75 h for TAP-B), and reduced pethidine consumption (110.40 ± 12.74 mg in 24 h). The study concluded that ESP-B provides more effective and longer-lasting pain relief compared to TAP-B.

In Alotaibi et al.’s randomized controlled trial of 60 lipoabdominoplasty patients, researchers evaluated the effectiveness of ultrasound-guided bilateral TAP-B for postoperative pain management [30]. Group A received the TAP-B, while Group B did not. Group A required significantly less opioid medication, had a longer time to the first analgesic request, and reported lower median VAS scores during mobilization. Fewer patients in Group A experienced nausea and vomiting. The study concluded that the ultrasound-guided TAP block effectively reduces pain and opioid consumption, recommending its use for improved patient outcomes in lipoabdominoplasty.

### 3.6. Studies Investigating Pain Pumps Using Local Anesthetics in Patients Who Underwent Rectus Plication

Mentz et al. examined the effectiveness of PPCs against standard pain medications. Their findings suggest that PPCs are highly effective in managing pain and reducing narcotic use in abdominal wall reconstruction, indicating a promising future for their routine use in post-abdominoplasty care [17].

### 3.7. Studies Investigating Pain Pumps Using Local Anesthetics in Patients Without Rectus Plication

Giordano et al. studied the efficacy of PPCs using bupivacaine or ropivacaine versus conventional analgesics in abdominoplasty but were limited by insufficient details on patient treatment. Despite this, they advocated for PPCs, highlighting their role in improving postoperative pain management, reducing opioid use, and decreasing hospital stays [21].

Bray Jr. et al., however, offered a more cautious view, suggesting that PPCs do not significantly improve postoperative pain control in abdominoplasty patients, thus highlighting the need for further research before their standardization in clinical practice [25].

Chavez-Abraham et al. demonstrated that elastomeric continuous-infusion pumps (ECIPs) substantially mitigated postoperative pain and reduced requirements for oral narcotics in elective augmentation mammaplasty or abdominoplasty [10]. Notably, the research also introduces lidocaine as a viable alternative to bupivacaine in ECIPs, potentially enhancing patient recovery outcomes.

Collectively, these studies illuminate the growing potential of PPCs in augmenting postoperative recovery in abdominoplasty procedures. Nonetheless, the observed variability in outcomes and methodologies across these studies underscores the necessity for more comprehensive and standardized research. Such endeavors are essential to ascertain the efficacy of PPCs conclusively and to develop optimal usage protocols for improved patient care.

### 3.8. Studies Investigating Local Anesthetics Postoperatively in Patients Who Underwent Rectus Plication

In Beaton et al.’s study, the bupivacaine implant demonstrated superior pain relief to placebo collagen implants during the first 24 h post-operatively, though this difference was not significant at the 48 and 72 h marks [11]. Edwards et al. revealed that a single intraoperative dose of liposome bupivacaine in patients undergoing breast surgery and abdominoplasty consistently resulted in lower pain scores, decreased opioid usage, heightened patient satisfaction, and fewer opioid-related side effects, in comparison to previous clinical experiences [13]. Morales Jr. et al.’s investigation into abdominal field block injections with liposomal bupivacaine in abdominoplasty with rectus plication indicated a notable reduction in postoperative pain, decreased reliance on narcotics, and a faster return to normal activities, highlighting its potential as a standard practice in postoperative pain management [16].

### 3.9. Studies Investigating Local Anesthetics Postoperatively in Patients Without Rectus Plication

Kakagia et al.’s study on fleur-de-lys abdominoplasty found that both ropivacaine and levobupivacaine were effective in mitigating postoperative pain, though levobupivacaine exhibited greater efficacy in terms of intensity and duration of analgesia [32].

### 3.10. Studies Investigating Opioids in Patients Who Underwent Rectus Plication

Minkowitz et al. demonstrated that 50 mg IV tramadol was more effective than a placebo and comparable to 4 mg IV morphine in managing postoperative pain, while exhibiting fewer adverse events, indicating its potential as a preferred opioid alternative [6]. In the APOLLO-2 trial, Singla et al. observed that oliceridine effectively managed moderate to severe acute postoperative pain in abdominoplasty patients, with its 0.35 mg and 0.5 mg doses showing equivalence to morphine in analgesic effectiveness and a better profile in terms of respiratory and gastrointestinal side effects [20]. At higher doses, oliceridine resulted in fewer incidents of nausea and vomiting than morphine, suggesting its viability as an alternative in situations requiring IV opioids.

### 3.11. Ketamine

Ali et al. evaluated low-dose ketamine versus morphine in abdominoplasty patients, focusing on the required intra- and postoperative fentanyl doses and side effects. They concluded that both treatments are equally effective in providing analgesia without significant side effects like sedation or hallucinations [20].

Besides the study by Ali et al., two additional studies explored the use of ketamine, with none of them explicitly mentioning patients that underwent rectus plication. In one such study, Mansour et al. discovered that incorporating 0.5 mg/kg of ketamine into levobupivacaine for TAP blocks notably decreased postoperative pain. This reduction was indicated by lower scores on the visual analogue scale and a reduced requirement for rescue morphine [28]. This combination also prolonged the duration before the first request for rescue analgesia, enhancing overall analgesic efficacy compared to administering levobupivacaine alone [28]. Varas et al. interestingly administered ketamine with magnesium, which halved morphine use and delayed the first morphine request compared to using ketamine alone or saline as a placebo [23]. Control patients in the latter two groups experienced more pain and higher morphine consumption, with no significant difference in adverse effects among groups [23].

### 3.12. NSAIDs

No included studies examined patients that underwent rectus plication. Sun et al. discovered that celecoxib significantly reduced postoperative pain and opioid analgesic need, expedited bowel function recovery by one day, and enabled earlier resumption of normal activities by two days compared to placebo in major plastic surgery patients. Additionally, patient satisfaction and quality of recovery were notably higher with celecoxib, but its administration preoperatively showed no advantage over postoperative use alone [33]. Singla et al. revealed that the administration of meloxicam IV at a dose of 30 mg resulted in notable pain relief in individuals undergoing abdominoplasty [18]. This effect was substantiated by a reduction in the summed pain intensity difference over 24 h, improvements in various secondary endpoints, and a reduction in the need for opioid rescue medication [18]. Additionally, it was observed that meloxicam IV maintained a favorable safety profile, devoid of an elevated risk of adverse events typically associated with nonsteroidal anti-inflammatory drugs [18].

### 3.13. Anesthesia

In the RCT conducted by Metry et al., 200 participants undergoing abdominoplasty were allocated into two distinct groups: one receiving general anesthesia (100 patients) and the other spinal anesthesia (100 patients). Postoperative pain was one of the assessed outcomes, which was quantitatively measured using the VAS scores at 2, 4, 6, and 12 h post-op. They recorded that the spinal group required less nalbuphine postoperatively (13.68 ± 3.42 mg) than the general anesthesia group (18.83 ± 3.79 mg). However, interestingly, the former recorded higher VAS scores overall than the latter [40]. None of the participants seem to have undergone rectus plication.

### 3.14. Opioids vs. NSAIDs

Türkoğlu et al.’s RCT found that the efficacy of IV tramadol, IV ibuprofen, and their combination was evaluated for postoperative analgesia in abdominoplasty patients [41]. The study found no statistically significant difference in morphine use as rescue analgesia across groups. Notably, nausea rates were significantly lower in the ibuprofen group compared to both the tramadol group and the combined treatment group, suggesting that IV ibuprofen might offer a preferable side-effect profile. The study highlights the potential of tramadol and IV ibuprofen, alone or in combination, to effectively manage postoperative pain while potentially reducing opioid consumption and related side effects. This aligns with clinical guidelines advocating for multimodal analgesia to minimize opioid reliance and enhance patient recovery outcomes [1,24]. Again, there was no explicit mention of patients undergoing rectus plication.

### 3.15. Use of Adjuncts

Silva Filho et al.’s prospective, controlled, double-blind trial evaluated the efficacy of magnesium sulfate as an analgesic in total intravenous anesthesia for post-bariatric abdominoplasty surgery [45]. Fifty patients were randomly assigned to two groups: the remifentanil group (RG) and the magnesium sulfate group (MSG). Key outcomes included changes in systolic blood pressure (SBP) and opioid consumption. The results indicated that while the RG experienced a decrease in SBP post-intubation and skin incision, the MSG demonstrated an increase, highlighting a significant treatment–time interaction (*p* = 0.028). Although eight MSG patients (34.8%) required supplemental fentanyl, 64% achieved adequate analgesia with magnesium sulfate. Notably, magnesium sulfate’s use led to reduced intraoperative opioid needs and maintained hemodynamic stability, suggesting its effectiveness in multimodal analgesia. In contrast, RG patients showed higher propofol and ephedrine consumption due to remifentanil’s potent analgesic effects, raising questions about the balance between opioid reduction and increased anesthetic requirements.

### 3.16. Results of the Bias Assessment

In this study, most RCTs achieved a “low” overall risk of bias. However, a notable concern was the inadequate detail provided regarding the concealment of allocation and the measures taken to prevent deviations from intended interventions. Only two studies provided a clear description of their concealment methods [20,32], and none of the RCTs comprehensively outlined strategies to mitigate deviations from the planned interventions. Consideration and management of missing outcome data were also not consistently explored across all studies. Only two RCTs elaborated on their processes of accounting for missing data [6,40]. All twenty-one articles had little to no issues with the measurements of their outcomes, using appropriate measurement scales to assess the efficacy of analgesia. While it is assumed that all RCTs should have received a pre-registered protocol, six of the included RCTs failed to mention which approvals they had attained [12,15,21,23,25,27,30]. Most cohort studies exhibited an overall risk score categorized as “moderate” or higher based on the ROBINS-I bias assessment tool. Nine investigations neglected to address various confounding variables among participants [7,13,22,24,25,30,31,41,45]. Only three authors explicitly outlined inclusion/exclusion criteria, contributing to a risk of bias in the remaining studies [7,25,30]. While the majority provided detailed explanations of interventions used, ten studies omitted reporting any deviations from intended techniques or the supervision of protocol adherence [7,24,25,30,41]. Concerningly, all studies lacked documentation on the handling of missing outcome data. Despite attempts to measure multiple outcomes, the reliability and validity of measurement tools utilized were unclear due to the lack of information provided. Furthermore, a majority of studies failed to report adjustments for multiple comparisons, introducing potential for type I error in the selection of reported results. One particular study was found to have significant risk of selection bias in most measured domains due to the loss of 54 participants through follow-up [16].

### 3.17. GRADE Assessment


**Article**

**Risk of Bias**

**Inconsistency**

**Indirectness**

**Imprecision**

**Publication Bias**

**Overall**
Beaton et al., 2023 [11]**Low**: Randomized, double-blind, placebo-controlled design with proper randomization, blinding, and allocation concealment. Minimal risk of bias from study design and execution.**Low**: There is no indication of inconsistency. Outcomes are measured systematically using standardized scales (e.g., NPRS) at predefined time points.**Low**: The study directly addresses the population, intervention, and outcomes relevant to postsurgical pain in abdominoplasty.**Moderate**: While the sample size is well-powered for the primary endpoint (SPI24), the lower power for SPI48 (66%) raises slight concerns about precision for secondary outcomes.**Low**: The study was registered on ClinicalTrials.gov, and the design reduces the likelihood of publication bias.**High Quality** The study exhibits a low risk of bias, direct applicability to the research question, and strong consistency in methodology. While there is some imprecision for the secondary outcomes, this is not sufficient to downgrade the overall quality, resulting in a high-quality rating.Abo-Zeid et al., 2018 [12]**Low**: Prospective, double-blinded, and randomized design reduces bias, but the randomization is via a closed envelope method. Minimal risk of bias from study design and execution.**Low**: No evidence of unexplained variability between groups or within study outcomes, as appropriate statistical tests (ANOVA, Kruskal–Wallis) were used for analysis.**Low**: The study directly addresses the population of interest (patients undergoing abdominoplasty) and interventions (TAP-B, RSB, and SCI) relevant to the research question.**Low**: Sample size is adequately powered for primary outcomes, and the statistical methodology is sound, giving confidence in the precision of the results.**Low**: Registered trial with an IRB approval and clinical trial registration (NCT03077581) reduces the likelihood of publication bias.**High Quality** The study has a strong design and execution with low risk in most domains.Edwards et al., 2015 [13]**Moderate**: This is an observational study, which inherently has a higher risk of bias compared to randomized trials. Although the study followed Good Clinical Practice guidelines and had IRB approval, there is no control group.**Low**: The study seems to have consistent methodology across multiple sites with similar treatments and data collection methods, reducing inconsistency risks.**Low**: The study directly addresses the relevant population (patients undergoing breast and/or abdominoplasty surgeries) and the intervention (liposome bupivacaine), making it applicable to the research question.**Moderate**: Some imprecision arises from the observational design, which limits the ability to draw causal inferences. However, the study appears to include a reasonable sample size and appropriate outcome measures.**Low**: A multicenter study with adherence to Good Clinical Practice and detailed outcome reporting reduces the likelihood of publication bias.**Moderate Quality** The observational nature and lack of a control group introduces some bias, but the study is well conducted and follows rigorous standards, making it a moderate-quality study.Gardner et al., 2019 [14]**Moderate**: The study is randomized, which reduces bias; however, there is no mention of blinding, which could affect the results and reporting of pain and narcotic consumption.**Low**: The results appear consistent within the study; statistical significance is noted between the two groups for both time points, suggesting reliable outcomes.**Low**: The study directly addresses the population undergoing abdominoplasty and the specific intervention (TAP-B), making it relevant to the research question.**Moderate**: While statistically significant differences were found, the sample size (20 participants) is small, which may limit the generalizability of the results.**Low**: The study presents clear data, and its design as a prospective comparative study reduces concerns about publication bias.**Moderate Quality** The randomized design supports quality, but the lack of blinding and small sample size introduces limitations. The significant findings still suggest valuable insights.Fiala 2015 [15]**Moderate**: The study design includes random assignment of patients to either the TAP block or standard treatment, but there is no mention of blinding, which may introduce bias in pain reporting.**Low**: The results are consistent across both groups, with statistically significant differences noted in narcotic use and time to first request for pain medication.**Low**: The study directly addresses patients undergoing abdominoplasty and evaluates a specific intervention (TAP block) relevant to post-surgical pain management.**Moderate**: While the sample size (32 patients) is reasonable for a pilot study, the findings, though statistically significant, should be interpreted cautiously due to this size.**Low**: The study presents clear and relevant data, with a focus on a specific clinical question, reducing concerns about publication bias.**Moderate Quality** The pilot study design shows potential efficacy of the TAP block but is limited by the lack of blinding and relatively small sample size, which could affect reliability.Minkowitz et al., 2020 [6]**Low**: The study is a multicenter, randomized, double-blind trial, which minimizes bias and enhances the reliability of the findings.**Low**: The study design is consistent in methodology across multiple centers, and the results are expected to be reliable due to the controlled design.**Low**: The study directly addresses the population of interest (patients undergoing abdominoplasty) and compares relevant treatment options (IV tramadol vs. morphine vs. placebo).**Moderate**: While the planned sample size of 360 patients enhances power, the actual results may vary, and the specific outcomes need to be interpreted with caution until completed.**Low**: The study’s registration and adherence to ethical guidelines reduce concerns about publication bias, ensuring transparency and accountability.**High Quality** The phase 3 design, randomization, blinding, and multicenter approach strengthen the overall quality of evidence. The study aims to evaluate both efficacy and safety comprehensively.Morales Jr et al., 2013 [16]**Moderate**: The study is retrospective and relies on case record reviews, which introduces potential biases in data collection and reporting.**Low**: The use of standardized methods for administering liposomal bupivacaine and consistent evaluation criteria across patients helps maintain consistency in the results.**Low**: The study directly investigates the efficacy of liposomal bupivacaine in postoperative pain management for patients undergoing abdominoplasty, aligning with the objective.**Moderate**: While average pain scores and medication usage provide some insight, variability in individual responses may limit the robustness of findings, requiring further validation.**Low**: The findings are likely to contribute valuable information to the existing literature on postoperative pain management, indicating a low risk of publication bias.**Moderate Quality** The study provides important data on the effectiveness of liposomal bupivacaine for pain management, but the retrospective nature and small sample size limit its overall quality.Price et al., 2023 [7]**Moderate**: Although the study has institutional review board approval and includes a clear inclusion criterion, it is retrospective and may have biases in data collection and patient selection.**Low**: The study employs standardized protocols for pain management across both groups, contributing to consistent outcomes.**Low**: The study directly addresses the impact of a multimodal approach to pain management in abdominoplasty patients, aligning with the stated objective.**Moderate**: The sample size of 80 patients is adequate, but potential variability in responses to pain management protocols may affect the precision of the findings.**Low**: The study’s findings are likely to fill a gap in literature regarding multimodal pain management in surgical settings, indicating a low risk of publication bias.**Moderate Quality** The study contributes valuable insights into multimodal pain management in abdominoplasty; however, the retrospective nature and potential biases in data collection limit its overall quality.Mentz et al., 2005 [17]**Moderate**: The study employs a randomized design, which reduces bias, but with a small sample size (20 patients), there may still be concerns regarding the potential selection and reporting bias.**Low**: The intervention and control groups were clearly defined, with a consistent method of pain assessment and a standardized protocol for administering treatments.**Low**: The study directly investigates the use of a Stryker Pain Pump versus standard pain management, addressing the research question without any indirect measures.**High**: With only 20 patients, the sample size is small, increasing the likelihood of variability in results and decreasing statistical power, contributing to imprecision.**Low**: Given the novelty of the use of a Stryker Pain Pump in postoperative pain management for abdominoplasty, there is no clear indication of publication bias.**Moderate Quality** The study presents an innovative approach to pain management with well-documented methodology, but the small sample size and potential biases limit the overall confidence in the results.Singla et al., 2018 [18]**Low**: This is a randomized, double-blind, placebo-controlled, multicenter trial with well-defined protocols, reducing the likelihood of selection and performance bias. Adherence to Good Clinical Practices further minimizes bias.**Low**: The study uses a standardized surgical procedure and pain assessment method across multiple clinical sites, with consistent endpoints, reducing potential inconsistency in outcomes.**Low**: The study directly investigates the effect of IV meloxicam on postoperative pain control, addressing the research question without indirect or surrogate measures.**Moderate**: The sample size is powered at >80%, which is strong, but the assumption of an effect size of 0.40 may affect generalizability to broader populations. Exact patient numbers are not provided, affecting the precision.**Low**: As a phase 3 trial for a well-established drug (meloxicam), there is low risk of publication bias. The trial follows standardized protocols, decreasing selective reporting risks.**High Quality** The study design is robust with a clear methodology, randomization, double-blind structure, and appropriate statistical analyses, providing high confidence in the reliability of the results.Bjelland et al., 2019 [19]**Low**: The study is a triple-blinded RCT with randomization conducted using a concealed computerized block algorithm, reducing bias. Blinding was maintained for patients, personnel, and analysts. However, a single surgeon performed the procedures, which could introduce performance bias, though steps were taken to standardize techniques.**Low**: There is no mention of significant variability in the outcomes. Pain and opioid consumption were measured at multiple time points, and consistent methods of assessment (NRS, PCA, etc.) were used across the study. Variability in results was controlled through the use of well-defined protocols and statistical methods, but no external replication was reported.**Moderate**: The population (post-bariatric patients undergoing abdominoplasty) and interventions (ropivacaine vs placebo for pain control) are highly specific to this clinical setting. While generalizable within this context, broader applicability to other populations or procedures may be limited.**Moderate**: The sample size of 50 patients was relatively small, though justified by power analysis. While confidence intervals were reported, the relatively small sample may limit the precision of the results, especially in detecting smaller effect sizes. However, a sample size of 23 per group was calculated to be sufficient to detect clinically relevant differences.**Low**: There is no indication of selective outcome reporting. The study was pre-registered with EudraCT and clinicaltrials.gov, minimizing the risk of publication bias. However, the absence of multiple independent studies reduces the ability to fully assess publication bias.**Moderate Quality** The evidence quality is strong due to the robust design (RCT), triple blinding, and use of appropriate statistical analyses. Some limitations exist, such as small sample size and indirectness to broader populations, but the study was conducted with high methodological rigor.Ali et al., 2020 [20]**Low**: The study was randomized with a computer-generated number table. Treatment allocation was concealed. Double-blinded design, with patients and the anesthetist recording the data blinded to group assignment. Adequate blinding of data collectors and outcome assessors.**Low**: No conflicting or heterogeneous results across groups were reported. The study used standard statistical methods (unpaired t-test, Chi-square) without notable variability in outcomes.**Moderate**: The study population is relatively specific (ASA I and II, ages 18–50, undergoing elective abdominoplasty), which may limit the applicability to other populations or surgeries. The interventions (ketamine vs. morphine) are widely used in clinical practice.**Low**: Power analysis was appropriately conducted, requiring 71 patients per group to achieve sufficient power. A total of 160 patients were enrolled to account for dropouts. Statistical analysis methods were adequately applied with sufficient patient numbers.**Low**: The study was registered (NCT03664622), followed transparent procedures, and reported conventional outcomes for pain management trials.
**High Quality**
Giordano et al., 2020 [21]**High**: As a retrospective study with no randomization, there is a higher risk of bias. The reliance on existing records and potential unmeasured confounders also increases bias.**Low**: The results were consistent across groups, with clear differences in opioid use and LOS, and similar complication rates. No notable variability was observed.**Moderate**: The population and interventions are relevant, but the small sample size and single-center design limit the generalizability to broader populations.**Moderate**: Despite significant findings, the small sample size and wide standard deviations introduce imprecision, reducing the reliability of the results.**Low**: There is no strong evidence of publication bias, as both significant and non-significant outcomes were reported.**Low Quality** This is due to the retrospective design and imprecision from the small sample size. However, the results are consistent and directly relevant to the study population.Shauly et al., 2022 [22]**High**: The study is retrospective with no control group or randomization, increasing the likelihood of bias, particularly selection and reporting bias. Additionally, all surgeries were performed by a single surgeon, which could introduce performance bias.**Moderate**: There is no clear mention of results variability or heterogeneity across patients, but the uniform protocol may reduce variability, indicating consistent outcomes. However, without comparative data, it is difficult to fully assess this.**Moderate**: The study population and interventions are relevant, but the highly specific surgical protocol and single-surgeon setting limit the generalizability to other contexts or surgeons.**Moderate**: The relatively small sample size of 80 patients and reliance on subjective pain scores without long-term follow-up data introduce imprecision in the estimates of effectiveness and safety outcomes.**Low**: There is no evidence of selective reporting, but the retrospective design and inclusion of a single surgeon’s patients may raise concerns about unpublished negative outcomes.**Low Quality** This is due to the retrospective, non-randomized design and moderate imprecision due to the small sample size and subjective outcome measures. While the outcomes are directly relevant, the highly specific surgical setting and protocol further reduce the generalizability of the findings.Varas et al., 2020 [23]**Moderate**: The study was randomized and double-blind, reducing selection and performance bias. However, the abandonment of the primary outcome after recruiting 25 patients raises concerns about reporting bias.**Low**: The methods for randomization and blinding were clearly outlined, and the interventions were well-defined, suggesting consistency. Additional analyses using both linear and nonlinear models further support robustness.**Low**: The study’s population (adults undergoing abdominoplasty/liposuction) and interventions (ketamine and magnesium) are relevant to clinical practice, limiting indirectness.**Moderate**: While a sample size calculation was performed, the outcome of interest was changed midway through the trial, which may lead to uncertainties in the findings related to morphine consumption.**High**: The study adhered to ethical guidelines and Good Clinical Practice. It was registered, ensuring transparency. The careful monitoring of adverse effects adds to the overall quality.**Low Quality** The randomized and double-blind design strengthens the study but concerns regarding the changes in the primary outcome and potential biases affect the overall confidence in the results.Silva Filho et al., 2021 [24]**Moderate**: The results indicate that the trial followed the CONSORT guidelines, which helps to ensure transparency in reporting participant flow. However, there were some missing blood concentration measurements due to sample mishandling, reducing the completeness of the dataset.**Low**: The study involves a single population with consistent methodology, inconsistency is unlikely**Low**: The findings for systolic blood pressure and secondary outcomes, such as fentanyl and ephedrine consumption, are consistent and follow logical trends between the two groups**Moderate**: There are some concerns regarding the precision of certain results. For instance, the study’s statistical significance was noted in certain comparisons (e.g., systolic blood pressure over time) with moderate effect sizes. However, the confidence intervals, especially for blood pressure post-intubation, were relatively wide.**Low**: There is no strong indication of publication bias based on the available data. The study appears to have been designed and reported transparently, without selective reporting of outcomes that would raise concerns of bias.**Moderate Quality** The evidence is reliable, though there are some concerns about sample size and imprecision in certain measures. However, the results appear consistent and relevant, providing useful insights into the effects of remifentanil and magnesium sulfate.Bray Jr et al., 2007 [25]**High**: The study is retrospective in nature, which inherently carries a risk of bias due to non-randomized group allocation. This can introduce selection bias, as patients may have been chosen based on factors not controlled for. Additionally, there was no blinding of patients or assessors, which could influence outcomes like pain reporting.**Low**: The outcomes showed small, non-statistically significant differences in all key measures, including pain medication use, pain scores, and hospital stay length. The direction of these small differences was consistent, suggesting that there is little unexplained variability between study results.**Low**: The population (abdominoplasty patients) and interventions (use of pain pumps) are directly relevant to the clinical question. There is no indirectness in terms of population, intervention, or outcomes that would limit the applicability of the results to similar clinical settings.**Serious**: The results did not achieve statistical significance for any outcomes, which limits the precision of the effect estimates. Additionally, the relatively small sample size (38 with pain pumps and 35 without) means that the study may not have had enough power to detect clinically meaningful differences.**Moderate**: As this is a single study, the risk of bias cannot be completely ruled out without evidence from other similar studies.**Low Quality** While the study addresses a relevant clinical question, the retrospective design, lack of blinding, and imprecision of results reduce confidence in the findings. The evidence provided is of low quality, meaning that further research is likely to impact the confidence in the estimate of effect and may change the findings.Chavez-Abraham et al., 2011 [10]**Serious**: This was a consecutive case series comparing patients before and after the use of an elastomeric continuous infusion pump (ECIP), without randomization. Lack of randomization and blinding raises the risk of bias, as the study groups were historical controls rather than being directly randomized. Postoperative pain and narcotic use are subjective, and the absence of blinding could lead to bias in reporting.**Moderate**: The study does not report significant variability across the different outcomes (pain scores and narcotic use), but without detailed statistical analysis, it is unclear whether the results are consistent across various subgroups or across different outcome measures. As the data are observational, some inconsistency could arise.**Low**: The population (augmentation mammaplasty and abdominoplasty patients) and the intervention (ECIP with lidocaine) are directly applicable to the research question on postoperative pain management in plastic surgery. The outcomes of interest (pain scores and narcotic use) are also clinically relevant.**Moderate**: The study involves a large number of patients (675 augmentation mammaplasty patients and 200 abdominoplasty patients in the control group; 690 and 215 in the intervention group). However, the results for pain scores and narcotic use are not reported with enough detail (e.g., confidence intervals or p-values) to fully assess precision. Given the large sample size, imprecision is likely not a significant concern.**Moderate**: The study does not directly provide evidence of publication bias, but publication bias could be a concern, particularly in observational studies where positive or favorable results are more likely to be published. Additionally, given the widespread interest in pain management innovations like ECIP, there may be a tendency to publish studies that report positive outcomes, even when the findings are not robust. However, there is no specific indication from the study design itself to suggest selective reporting or significant bias.**Low Quality** While the study includes a large sample size and directly relevant patient population, the lack of randomization and blinding introduces bias. Additionally, the results are not presented with sufficient statistical rigor to assess precision fully, though the sample size somewhat mitigates this concern.Singla et al., 2019 [26]**Low**: The study was a phase III, multicenter, randomized, double-blind, placebo- and active-controlled trial, which is a robust and high-quality design for clinical trials. Randomization and blinding were performed, and the use of placebo and active comparators strengthens internal validity.**Moderate**: The use of clinician-administered doses and the possibility of protocol deviations during the study may introduce some performance bias. In addition, while pain intensity and respiratory events were measured objectively, the interpretation of some outcomes (like respiratory safety events) may be subject to observer bias, as they depend on clinical judgment. Overall, though, the risk of bias appears minimal.**Low**: The population and interventions are well-aligned with the clinical question of interest: pain management in post-surgical patients undergoing abdominoplasty. There are no significant issues with indirectness, as the study used appropriate comparators (morphine and placebo) and measured clinically relevant outcomes (pain intensity, respiratory safety). The study is directly applicable to the clinical scenario it seeks to address.**Low**: The sample size (375 patients) was calculated to provide high statistical power (>88%) for key endpoints, such as superiority to placebo and noninferiority to morphine. The study design and power calculations suggest that imprecision is not a significant concern, as sufficient numbers were included to detect meaningful differences.**Low**: As the study is part of a well-documented clinical trial with publicly available registration (NCT02820324) and conducted in a rigorous phase III setting, there is no evidence to suggest publication bias. However, as always with industry-sponsored trials, there is a potential risk that negative results from similar trials may not be published. Nonetheless, no direct evidence of publication bias is indicated here.**High Quality** This study design and conduct were strong, with minimal risks of bias, indirectness, and imprecision. The high level of evidence suggests reliable conclusions can be drawn from the study’s findings.Elsawy et al., 2021 [27]**Moderate**: The study is single-blinded, which may introduce bias in patient-reported outcomes. Randomization was present, but allocation concealment was not clearly mentioned.**Low**: The results showed consistent findings with significantly lower VAS scores and prolonged analgesic effects in the ESP group across multiple time points.**Low**: The population, intervention, and outcomes are directly relevant to the clinical question. The study population was specifically patients undergoing abdominoplasty.**Moderate**: While the results are statistically significant, the sample size (51 patients) may not provide robust generalizability, and confidence intervals around the estimates were not reported.**Low**: There is no indication of publication bias based on the outcomes; however, this cannot be definitively assessed without a systematic review of all available literature.**Moderate Quality** Given the moderate risk of bias, moderate imprecision, and strong consistency in results, the overall quality of evidence is rated as moderate.Mansour et al., 2021 [28]**Low**: The study is double-blinded and randomized, reducing the risk of bias significantly. The randomization process was clearly described, and informed consent was obtained.**Low**: The outcomes were consistent across different time points and groups, with clear comparisons between the TAP and LK groups, showing statistically significant differences.**Low**: The study population and interventions are directly relevant to the research question regarding pain management in abdominoplasty patients, with appropriate outcomes measured.**Moderate**: While the sample size was adequate (50 patients), confidence intervals and specific p-values were not reported, which could affect the reliability of the results.Low: There are no apparent indicators of publication bias, and the study’s outcomes seem to have been reported transparently.**High Quality** The overall quality of evidence is moderate, reflecting low risk of bias, low inconsistency, and low indirectness, but moderate imprecision due to lack of detailed statistical reporting.Salama et al., 2018 [29]**Moderate**: The study employed randomization and blinding for patients and outcome assessors, which helps reduce bias. However, the lack of blinding for the surgeon performing the procedure introduces potential bias in treatment administration and outcome assessment.**Low**: The study appears consistent in its methodology and outcomes across the three groups. There were clear protocols for each intervention, and the outcomes measured (morphine consumption, NRS scores, etc.) are well-defined and comparable.**Low**: The study population and interventions are directly relevant to the clinical question being addressed (pain management in patients undergoing abdominoplasty). The inclusion criteria ensure a focused patient group, minimizing indirectness in the applicability of results.**Moderate**: While the sample size of 90 patients is reasonable for detecting differences, some outcomes may have wide confidence intervals due to variability in individual patient responses. Further details on the specific effect sizes and confidence intervals would enhance precision.**Low**: The study does not indicate signs of publication bias, and prospective randomization suggests a lower likelihood of selective reporting. However, the assessment is limited due to the absence of systematic searches for unpublished studies or protocols to evaluate bias thoroughly.**Moderate Quality** The overall quality of evidence is moderate, given the sound methodology, reasonable sample size, and clear outcomes. However, the potential for risk of bias from the unblinded surgical procedure and some concerns regarding imprecision may affect the robustness of the conclusions drawn from the study.Alotaibi et al., 2020 [30]**Low**: The study is a randomized controlled trial with proper blinding and handling of dropouts and withdrawals**Low**: The findings are consistent within the study. The outcomes indicate a clear distinction in pain management between the two groups, with a significant difference in opioid consumption and VAS scores. There is no indication of variability that would suggest inconsistency across similar studies.**Low**: The study directly addresses the population of interest (patients undergoing lipoabdominoplasty) and the intervention (ultrasound-guided TAP block). This makes the evidence highly relevant and minimizes concerns about indirectness.**Moderate**: While the sample size of 60 patients is reasonable, the findings regarding opioid consumption and pain scores could be subject to variability, especially if confidence intervals and effect sizes are not provided. This indicates a moderate level of imprecision in the estimates of the treatment effect.**Low**: There is no indication of publication bias, as the study addresses a relevant clinical question and reports significant findings.
**High Quality**
Farahat et al., 2023 [31]**Moderate**: The study likely involved random allocation of patients to groups. However, potential biases in allocation concealment or blinding may exist, affecting the validity of results.**Low**: Results between the groups (ESP-B and TAP-B vs. control) are consistent in terms of reduced opioid consumption and improved analgesia duration, suggesting low inconsistency.**Low**: The study directly addresses the analgesic efficacy in the specific population (females undergoing abdominoplasty) and relevant interventions (ESP-B and TAP-B).**Moderate**: While the study presents clear results, the sample size of 69 may limit the precision of the estimates, especially regarding rare events or specific outcomes.**Low**: There is no indication of publication bias, as the study reports outcomes clearly and is likely to be part of the ongoing literature on multimodal analgesia.**Moderate Quality** Considering the moderate risk of bias and imprecision, the overall quality of evidence is rated as moderate, indicating that further studies may be necessary to strengthen the findings.Kakagia et al., 2007 [32]**Low**: The study uses appropriate randomization and blinding**Low**: The results from different groups (saline, ropivacaine, levobupivacaine) seem consistent, assuming that the comparisons made are appropriate. No significant discrepancies in findings were mentioned.**Low**: The study population appears directly relevant to the clinical question, as all patients underwent mini abdominoplasty, which is the focus of the investigation.**Moderate**: While the sample size of 46 patients is reasonable, results could be imprecise if the confidence intervals of the outcomes are wide or not reported.**Low**: No indication of publication bias is provided. The study seems to report its findings transparently. However, further data about the availability of studies could provide a clearer picture.**High Quality** The study provides relevant insights into postoperative analgesia following mini abdominoplastySun et al., 2008 [33]**Low**: The study has a well-defined randomization process and blinding for participants and staff**Low**: The study appears consistent in its findings across treatment groups, with no conflicting results reported within the design or outcomes assessed.**Low**: The study population is relevant to the research question, focusing on patients undergoing major plastic surgeries, which aligns well with the objectives of the study.**Low**: The sample size is adequately large, with 120 participants in total**Low**: There are no indications of publication bias in the methods or findings reported. The study seems to transparently report all relevant outcomes.**High Quality** The methods employed are generally sound and appropriate for the study objectives.Michaels et al., 2009 [34]**Moderate**: While the study involves a retrospective review of cases with a defined senior author, there is no mention of randomization and potential selection bias in grouping patients may affect the reliability of results.**Low**: The outcomes reported for rib blocks versus general anesthesia show consistent results across the measures assessed (recovery room time, narcotics, nausea/vomiting, pain), with significant differences noted.**Low**: The study population is relevant to the surgical context being examined, focusing specifically on abdominoplasty patients, allowing for direct application of findings.**Moderate**: The sample sizes (39 in group 1 and 29 in group 2) are relatively small, which could affect the precision of the estimates. Detailed confidence intervals for the significant results were not provided, limiting interpretability.**Low**: There is no indication of publication bias. The results appear to be reported transparently, with no selective outcome reporting evident.**Moderate Quality** The overall quality is moderate, as the study employs a clear methodology and demonstrates significant findings; however, limitations in study design and sample size warrant cautious interpretation.Gravante et al., 2011 [35]**Moderate**: The retrospective design introduces a potential for bias, particularly in patient selection and matching. While efforts were made to match groups for age and sex, residual confounding may still be present due to the nature of retrospective data.**Low**: The study provides consistent results regarding postoperative pain management across the two groups, with clear outcomes related to analgesic requirements.**Low**: The study population is relevant to the research question, focusing specifically on patients undergoing abdominoplasty with TAP blocks, allowing direct applicability of findings.**Moderate**: The sample size of 51 patients is relatively small, which can reduce the precision of the results**Low**: There are no indications of publication bias, as the study reports outcomes comprehensively and focuses on relevant measures.**Moderate** Overall quality remains moderate due to the retrospective nature of the study and potential biases, but the clarity and consistency of results strengthen the findings.Meouchy et al., 2021 [36]**Low**: The study used random allocation to assign patients to groups, which minimizes selection bias.**Low**: The results are consistent, with significant differences noted in multiple outcomes (morphine and tramadol consumption, pain severity, and quality of recovery) between the two groups.**Low**: The study’s population and interventions are directly relevant to the question posed, focusing on postoperative analgesia in patients receiving quadratus lumborum blocks.**Low**: Results are statistically significant, with clear mean values and confidence in the measured outcomes, indicating low variability in the data.**Low**: There are no indications of publication bias, as the study presents clear and relevant outcomes, and there are no conflicts of interest mentioned.**High Quality** The overall quality of the evidence is high due to the low risk of bias, consistency, and clear outcomes measured.Sforza et al., 2024 [37]**Low**: The study design is randomized and double-blind, reducing the risk of selection and performance bias. All participants had similar characteristics, and allocation concealment appears well-managed.**Low**: The results are consistent across multiple time points, showing significant differences in pain scores and morphine requirements between the PECS and no-PECS groups.**Low**: The study population and intervention are directly relevant to the research question regarding analgesia in breast augmentation surgery.**Low**: Results are statistically significant with precise mean values and standard deviations provided for primary outcomes, indicating low variability in the findings.**Low**: There are no indications of publication bias, as the study reports clear and relevant outcomes, and the protocol was approved by an ethical board.**High Quality** The overall quality of evidence is high, with low risk of bias, consistency, directness, and precision in the findings.Feng 2010 [3]**Moderate**: The study is retrospective and lacks randomization, which introduces potential selection bias. However, it uses a control group and evaluates a large sample size, which partially mitigates this concern.**Low**: The results are consistent across different severity classes, showing significantly less pain and reduced narcotic use in the treatment group compared to the control group.**Low**: The patient population and intervention are directly relevant to the question of pain management in abdominoplasty procedures.**Moderate**: While the treatment effects are significant, the lack of detailed statistical analysis on variability (e.g., confidence intervals) limits the precision of the findings.**Low**: There is no indication of publication bias, as the outcomes are clear and relevant, with a focus on clinical efficacy based on patient questionnaires.**Moderate Quality** Overall, the study provides moderate-quality evidence due to the retrospective design and potential biases, despite strong findings regarding pain reduction and recovery.Araco et al., 2010 [38]**Moderate**: The study is observational with potential for selection bias based on classification into TAP+ and TAP- groups.**Low**: The results show consistent findings, with TAP+ patients significantly requiring less analgesia, indicating no significant variation in outcomes across the study population.**Low**: The patient population and interventions evaluated directly address the question of TAP block efficacy in postoperative pain management after surgery.**Moderate**: While the results indicate a significant reduction in analgesic requirements, the lack of detailed statistical measures (e.g., confidence intervals) limits the precision of the findings.**Low**: There is no clear indication of publication bias as the outcomes reported are straightforward and based on clear clinical efficacy measures.**Moderate Quality** The evidence suggests a positive impact of TAP blocks on reducing analgesia requirements postoperatively, but the observational nature and potential biases warrant a moderate quality rating.Wu et al., 2019 [39]**Low**: The study is randomized and double-blinded, which minimizes selection and performance bias. Clear inclusion and exclusion criteria are well defined, enhancing the validity of the findings.**Low**: The results are consistent across the different groups, with no significant differences in VAS scores and consumption of dezocine, supporting the reliability of the outcomes.**Low**: The population, interventions, and outcomes directly relate to the research question. The study design is appropriate for assessing the efficacy of pain management techniques in LC.**Low**: The sample size of 180 patients provides a robust data set, and outcomes are clearly reported. There are no indications of wide confidence intervals or uncertain estimates affecting the results.**Low**: The study design and outcomes are straightforward and relevant to the clinical question, with no apparent indication of selective reporting or publication bias.**High Quality** Given the low risk of bias, low inconsistency, low indirectness, low imprecision, and low publication bias, the overall quality of the evidence is high, indicating strong support for the study’s conclusions.Metry et al., 2019 [40]**Moderate**: The study is randomized, but the blinding of participants and assessors is not explicitly stated. Selection bias is minimized through randomization, but without clear details on blinding, risk remains moderate. The exclusion criteria were also broad, which could introduce bias.**Low**: Results appear consistent across outcomes measured, with similar patterns of pain scores and recovery times reported in both groups. No significant variability in the results is noted.**Low**: The study directly addresses the clinical question of pain management techniques in abdominoplasty, using relevant outcomes such as VAS scores, total analgesic consumption, and patient satisfaction. The population is appropriate for the study’s aims.**Moderate**: The sample size of 200 patients provides a reasonable power for detecting differences; however, specific confidence intervals or standard deviations for key outcomes were not provided, making precise interpretations challenging.**Low**: The study is registered with a clinical trials registry, indicating an intent to publish, and reducing concerns about selective reporting. The outcomes seem to align with the registered protocol.**Moderate Quality** Given the moderate risk of bias, low inconsistency, low indirectness, moderate imprecision, and low publication bias, the overall quality of evidence is moderate, suggesting the findings can be considered reliable but with some caution.Türkoglu et al., 2022 [41]**Moderate**: The study design appears to involve randomization, but details about allocation concealment and blinding of participants and assessors are not provided, leaving potential for bias. The reporting of patient selection and any exclusions is also unclear, impacting the assessment of bias.**Low**: The results show consistent findings regarding pain control across the three groups, with significant differences in VAS scores and analgesic consumption. No discrepancies in outcomes are noted, suggesting low inconsistency.**Low**: The study directly addresses the clinical question of postoperative pain management in abdominoplasty patients, making the findings applicable to the target population and relevant outcomes.**Moderate**: While the study provides significant results with clear differences in pain scores and opioid consumption, specific confidence intervals or standard deviations for the key outcomes are not mentioned, which can hinder precise interpretation.**Low**: The study presents results with clear outcomes, and there is no indication of selective reporting. Given the straightforward nature of the intervention and outcomes, publication bias is unlikely.**Moderate Quality** The overall quality of evidence is moderate, considering the moderate risk of bias, low inconsistency, low indirectness, moderate imprecision, and low publication bias.

### 3.18. Limitations

A significant challenge lies in the heterogeneity of surgical procedures across the studies. Many studies pooled together different types of surgeries, ranging from abdominoplasties to breast reconstructions, without adequately considering the specific pain profiles and postoperative recovery requirements unique to each procedure. This lack of specificity reduces the ability to tailor analgesic interventions effectively. For example, studies like Shauly et al. and Morales Jr. et al., while focused on specific interventions, did not control for variations in surgical technique, incision size, or the extent of tissue manipulation, all of which can dramatically influence postoperative pain levels. Consequently, the substantial heterogeneity across studies precluded the possibility of conducting a meta-analysis, as it could compromise the validity and interpretability of the results.

Moreover, the duration of follow-up in most studies was insufficient to fully assess long-term outcomes related to pain management. While many studies measured pain scores in the immediate postoperative period, few extended beyond the first 72 h post-surgery. This narrow time frame fails to capture the full spectrum of patient recovery and the potential development of chronic pain, a crucial endpoint in evaluating the efficacy of analgesic interventions. For instance, Edwards et al. reported effective pain relief with a specific intervention in the short term, but the absence of long-term follow-up data prevents understanding whether this benefit persisted or if it contributed to better patient outcomes weeks after surgery.

A further limitation involves the use of inconsistent pain assessment tools across studies. The reliance on subjective measures such as the VAS or numerical rating scale (NRS) without standardization complicates comparisons between studies. While these tools are widely used, the lack of uniform timing for pain assessments (e.g., at rest, during movement) introduces variability. Some studies assessed pain only at rest, while others incorporated dynamic pain measures, yet no consensus exists on which is the most appropriate or clinically relevant endpoint. Consequently, this inconsistency in pain measurement creates difficulties in synthesizing data across the review.

In addition to variations in pain assessment, opioid consumption, which is a critical outcome of postoperative analgesia, was not consistently reported across studies. Some studies quantified opioid use in morphine milligram equivalents, while others merely reported reductions in usage without precise metrics, limiting the comparability of opioid-sparing effects. For example, while Gardner et al. documented significant reductions in opioid consumption with TAP-Bs, other studies either failed to measure opioid use directly or used different units of measurement, making it difficult to establish a clear understanding of the clinical benefit in reducing opioid reliance.

Lastly, many studies failed to account for patient-related factors such as psychological distress, pre-existing chronic pain, or opioid tolerance, all of which can influence postoperative pain perception and analgesic requirements. Without controlling for these variables, it becomes challenging to attribute differences in outcomes solely to the analgesic interventions being studied. For example, patients with high levels of preoperative anxiety or those with a history of opioid use disorder might require more intensive pain management, skewing the results in favor of more aggressive analgesic techniques. Future research should aim to stratify patients based on these factors to provide more nuanced and reliable recommendations for clinical practice.

## 4. Discussion

The increasing prevalence of abdominoplasty over the past decade reflects not only heightened interest in cosmetic enhancement but also advancements in surgical techniques that promise safer outcomes and more effective recoveries [1]. This systematic review delves into the realm of postoperative pain management following abdominoplasty, a critical aspect that significantly influences patient recovery and satisfaction.

Abdominoplasty is one of the most undertaken elective surgeries, necessitating meticulous preoperative planning to ensure successful outcomes. Effective intraoperative and postoperative pain management can not only enhance patient well-being, but also expedite patient mobilization, and reduce hospital stays and associated costs [5,26,34,35,46]. The traditional reliance on narcotics, such as morphine, raises concerns in the wake of the escalating opioid crisis. The past two decades have seen a dramatic increase in opioid prescriptions without a corresponding rise in reported pain, leading to a surge in addiction and overdose-related deaths [47,48,49,50,51]. Moreover, plastic surgeons often prescribe opioids in excess, leaving patients with unused medication that can be misused or inappropriately disposed of [52]. To mitigate this, surgeons must adopt more judicious prescribing practices, prioritize alternative pain management techniques, and educate patients on the risks of opioid misuse. Additionally, implementing strict protocols for opioid prescription, such as prescribing the minimum effective dose and duration, and ensuring proper disposal of unused medications, are crucial steps in curtailing the risks associated with opioid use in postoperative care.

The undesirable side effects of opioids, such as sedation, nausea, and constipation, further complicate postoperative recovery, potentially prolonging hospital stays and increasing healthcare costs [53,54,55,56,57]. Thus, there is a pressing need for plastic and aesthetic surgeons to consider alternative analgesic modalities that can mitigate postoperative pain while minimizing opioid use. Studies have shown the efficacy of nonopioid analgesics in reducing the need for morphine, thereby lowering pain levels and enhancing patient satisfaction [58,59,60,61]. The adoption of nonopioid analgesics in practice is therefore crucial to address these issues, offering a more balanced and patient-friendly approach to pain management in aesthetic procedures.

Particularly noteworthy is the role of TAP blocks in reducing morphine intake, overall postoperative pain [12,14,15,20,29,41], and reduced adverse effects typically caused by opioids like nausea and vomiting. Patients receiving TAP blocks had longer times to requiring first analgesia compared to RS-Bs and Sci [12]. This, coupled with greater patient satisfaction with pain control than the controls, positions TAP blocks as highly promising alternatives. However, the application of TAP blocks is not without its limitations and potential complications, including cost considerations and risks of liver injury, peritonitis, vascular injury, and transient nerve palsies in certain cases [62,63]. Additionally, the optimal dosing of bupivacaine for TAP blocks remains undetermined [64]. Obesity also plays a significant role in abdominoplasty outcomes, influencing both the need for analgesia and the safety of opioid use [5]. Obesity is linked to both the outcomes of opioid overdose hospitalizations and the likelihood of using strong opioids [62]. Obese patients hospitalized due to opioid overdose tend to experience more severe complications like respiratory failure and require more hospital resources, yet show lower in-hospital mortality rates, indicating a need for further research on this ‘obesity paradox’ [65]. Additionally, the risk of using strong opioids increases with obesity severity; individuals with mild, moderate, and severe obesity are 1.31, 1.73, and 2.44 times more likely, respectively, to use these opioids compared to those with a normal BMI [66]. Stokes et al. demonstrate a clear correlation between increasing levels of obesity and a higher likelihood of receiving prescription opioids, with overweight and obese individuals comprising over 16% of opioid prescription recipients [67]. This underscores the need for plastic and aesthetic surgeons to carefully evaluate pain management options, particularly concerning obesity and its impact on opioid-related adverse effects that can complicate the postoperative recovery phase.

This systematic review critically evaluates the methodological and outcome biases across various studies, highlighting the imperative need for standardized research protocols to bolster the credibility of conclusions drawn. The review also points out prevalent issues such as publication and selection biases, along with the inconsistent use of pain assessment scales and reporting of patient satisfaction in existing literature. While this research offers significant insights into alternative approaches for managing postoperative pain, it is crucial to recognize its inherent limitations. The study presents a notable heterogeneity in participant selection, variations in adherence to the intended interventions, challenges in managing missing data, and diversity in outcome measurements. Of the 12 analyzed studies, only three clearly outline their participant inclusion criteria. Concerns about sample sizes are prominent, as seen in Gardner et al.’s study involving only 20 women aged 25 to 65, which may lead to cognitive bias due to its limited representation of the general population. Similar limitations are observed in Giordano et al.’s study with 61 participants. Additionally, nine studies report insufficient information on deviations from planned procedures and adherence to protocols. For instance, Price et al. allowed the control group to choose non-opioid analgesics, while restricting the experimental group to a specific set of analgesics, thereby introducing variability in the intervention. The handling of missing data is another critical issue in all 12 studies, exemplified by Morales Jr.’s exclusion of 54 participants due to loss of follow-up or inability to provide accurate information, without addressing how this affects the results. Moreover, eight studies lack clarity in their measurement tools, which raises concerns about the reliability and validity of the outcomes. Morales Jr., for instance, used a subjective 1–10 pain scale, potentially introducing bias and confounding variables. These limitations underline the necessity for enhanced methodological stringency and standardized reporting in future research. To ensure reliable and generalizable findings, future studies should adopt stricter participant selection criteria and maintain consistent intervention protocols. The management of missing data needs thorough attention to uphold the integrity and validity of the research. Additionally, the use of clear and objective measurement tools is crucial to minimize biases and enhance the robustness of the results. Advancements in research methodology and reporting standards are vital for progressing in the field of postoperative pain management. Such improvements will lead to more efficacious and safer treatment protocols that can be uniformly implemented across various patient demographics.

This review offers a comprehensive evaluation of both opioid and non-opioid interventions for pain management following abdominoplasty, highlighting the growing need to reduce opioid reliance in the context of the current opioid crisis. It underscores the variability in study designs and the heterogeneity of surgical procedures, which present challenges in standardizing pain management protocols. Despite these limitations, the review identifies significant advantages in exploring non-opioid analgesics and locoregional anesthesia techniques, particularly the use of TAP-B, which have shown promise in minimizing opioid use and improving patient outcomes. Additionally, the review brings attention to the underexplored impact of obesity on opioid-related complications in abdominoplasty patients, offering new insights and avenues for future research in postoperative pain management.

Ultimately, the authors recommend that future approaches to postoperative pain management in abdominoplasty focus on a combination of individualized patient care and multimodal analgesic techniques. Surgeons should integrate both pharmacologic and non-pharmacologic strategies, considering factors such as patient demographics, pre-existing conditions, and obesity. Non-opioid alternatives, like local anesthetic techniques and NSAIDs, should be prioritized to reduce opioid reliance and minimize adverse effects. Additionally, the development of standardized protocols for pain management, participant selection, and outcome measurement is critical to enhance research reliability and clinical application. Educational efforts should also focus on patient awareness regarding the risks of opioids, ensuring proper use, prescription, and disposal practices to mitigate long-term addiction risks. Future research should aim to clarify optimal dosing regimens, particularly for local anesthetics, and further investigate the impact of obesity on both pain management needs and recovery outcomes, with an emphasis on reducing complications [68,69].

## 5. Conclusions

The potential of nonopioid analgesics as substitutes for morphine in the management of postoperative pain among abdominoplasty patients is apparent, yet further investigation is necessary to systematically assess their efficacy. Future research endeavors should emphasize conducting RCTs encompassing large and diverse participant groups. This approach is crucial for adequately representing the variance in patient demographics and surgical methodologies. Additionally, the employment of uniform and standardized metrics for evaluating postoperative pain is of paramount importance. Such standardization is key to reducing bias and enabling more effective meta-analytical studies. Achieving these research objectives will significantly contribute to the development of more efficient and safer pain management protocols for abdominoplasty patients, thereby enhancing patient outcomes and optimizing healthcare resources.

## Figures and Tables

**Table 1:** Features of the included studies that discuss rectus plication or diastasis correction.

Article Number	Author and Year	Methodology	Sample Size (Abdominoplasties Only)	Age Mean/Range	Intervention(s) Used	Outcome(s)
1	Beaton et al., 2023 [11]	RCT	365 (181 bupivacaine, 184 placebo)	18–65	Bupivacaine implant vs. Placebo	Bupivacaine implant provided superior pain relief compared to placebo collagen implants in the first 24 h post-operation, with no significant difference at the 48 and 72 h marks
2	Abo-Zeid et al., 2018 [12]	RCT	48 (16 TAP-B, 16 RS-B, 16 SCI)	NIL	TAP-B vs. RS-B vs. Subcutaneous infiltration (SCI)	TAP-Bs outperform RS-Bs and Sci in prolonging analgesia and reducing morphine requirements
3	Edwards et al., 2015 [13]	Prospective	15	43 ± 13	Liposome bupivacaine	A single intraoperative dose of liposome bupivacaine in breast surgery and abdominoplasty patients yielded consistently lower pain, reduced opioids, greater satisfaction, and fewer side effects compared to past clinical outcomes
4	Gardner et al., 2019 [14]	Prospective	20 (10 RP, 10 TAP)	43.8 (TAP) 38.8 (RP) /25–65	Rectus plication (RP) block vs. Transverse abdominis plane (TAP) block	TAP-Bs prior to abdominoplasty significantly cut narcotic use, pain levels, and delayed pain medication requests, while also promoting early walking, proving their effectiveness as a post-surgical pain reliever.
5	Fiala 2015 [15]	Prospective	32 (16 standard, 16 TAP)	41.4 (standard) 44.8 (TAP)	Pararectus injections + ilioinguinal/iliohypogastric nerve blocks (standard) vs. TAP block	Patients receiving TAP blocks needed less postoperative hydromorphone and took longer to request pain medication, indicating more effective analgesia compared to standard nerve blocks after abdominoplasty
6	Minkowitz et al., 2020 [6]	RCT	370 (136 placebo, 141 tramadol, 93 morphine)	18–75	IV tramadol 50 mg vs. IV morphine 4 mg vs. Placebo	IV tramadol at 50 mg outperformed a placebo and matched 4 mg IV morphine in postoperative pain management with fewer side effects, suggesting it as a favored opioid alternative.
7	Morales Jr. et al., 2013 [16]	Retrospective	64	42/25–67	Liposomal bupivacaine	Abdominal field block injections with liposomal bupivacaine in abdominoplasty significantly reduced postoperative pain, narcotic use, and expedited return to normal activities, suggesting its standard use in postoperative pain management
8	Price et al., 2023 [7]	Retrospective	80 (42 control, 38 multimodal analgesia)	37.3 (control) 42.6 (multimodal)	Multimodal approach (refer to Table 1 in study)	Less narcotic use and better pain control with multimodal medications, suggesting a need for surgeons to revise prescribing practices to combat the opioid crisis
9	Mentz et al., 2005 [17]	RCT	20 (10 pain pump, standard pain meds)	NIL	Pain infusion pump (0.5% bupivacaine) vs. Standard pain meds	The group using a pain pump had significantly less postoperative pain, increased mobility, resumed activities sooner, and used fewer narcotics, suggesting its benefits may justify the cost in abdominal wall reconstruction
10	Singla et al., 2018 [18]	RCT	219 (110 meloxicam, 109 placebo)	38.9 ± 8.40 (melox) 41.0 ± 9.63 (placebo) /18–75	IV meloxicam 30 mg vs. Placebo	Meloxicam IV significantly improved pain relief and reduced opioid rescue medication use compared to placebo in abdominoplasty patients, with a safety profile similar to placebo and no increased risk of typical NSAID-related adverse events
11	Bjelland et al., 2019 [19]	RCT	50 (25 ropivacaine, 25 normal saline)	42 (Ropivacaine) 40 (normal saline) /18–64	Ropivacaine (3.75 mg/mL) vs. Normal saline (9 mg/mL) Both groups received a quadratus lumborum block	Quadratus lumborum block within multimodal pain management did not significantly reduce opioid needs or postoperative discomfort, with technical challenges encountered during administration and minimal observed benefit within the first 12 h postoperatively

**Table 2:** Features of the included studies that do not explicitly mention performing rectus plication or diastasis correction.

Article Number	Author and Year	Methodology	Sample Size (Abdominoplasties Only)	Age Mean/Range	Intervention(s) Used	Outcome(s)
1	Ali et al., 2020 [20]	RCT	160 total (80 with ketamine, 80 with morphine)	33.03 ± 6.14 (K) 32.23 ± 5.21 (M) /18–50	IV ketamine vs. IV morphine	Compared low-dose ketamine and morphine in abdominoplasty patients for analgesia efficacy and side effects, finding both equally effective without significant sedation or hallucinations
2	Giordano et al., 2020 [21]	Retrospective	61 (24 PPC, 37 CAA)	44.7 ± 9.1 (PPC) 40.5 ± 9.9 (CAA)	Pain pump catheter (PPC) vs. Conventional abdominoplasty analgesia (CAA)	The study favored PPCs with bupivacaine or ropivacaine for enhancing postoperative pain management and reducing opioid use and hospitalization in abdominoplasty
3	Shauly et al., 2022 [22]	Retrospective	80	42.88 ± 2.40/25–68	Liposomal bupivacaine intraop (within a pain protocol)	Patients undergoing lipoabdominoplasty with a multimodal analgesia protocol experienced low mean pain scores postoperatively, averaging 0.46/10 initially and 1.24/10 by day 7, with most switching to NSAIDs post-day 7 and nearly 94% reporting no pain at 4–6 weeks, highlighting the protocol’s effectiveness in pain management.
4	Varas et al., 2020 [23]	RCT	63 (21 control, 20 ket grp, 22 ket-mag)	40.2 ± 8.5 (control) 39.8 ± 6.1 (ket) 39.2 ± 7.2 (ket-mag)	IV 0.3 mg/kg ketamine + 50 mg/kg magnesium (KetMag)VS.Saline (control) vs. 1 bolus ketamine (ket grp)	Combining ketamine with magnesium significantly reduced morphine consumption and delayed its first request versus using ketamine alone or saline, without increasing adverse effects
5	Silva Filho et al., 2021 [24]	RCT	43 (20 RG, 23 MSG)	37.15 ± 10.33 (RG) 39.87 ± 6.57 (MSG)	Remifentanil group (RG) vs. Magnesium sulfate group (MSG)	Magnesium sulfate significantly reduced the need for additional analgesia and ephedrine while increasing propofol use, offering a safe and effective opioid-sparing option for intraoperative analgesia
6	Bray Jr. et al., 2007 [25]	Retrospective	73 (38 local anesthetic pump, 35 standard pain meds)	NIL	Pain infusion pump (bupivacaine) vs. Standard pain meds	The use of pain pumps in abdominoplasty patients led to a minor, nonsignificant reduction in pain medication use and no significant improvement in pain scores or hospital stay
7	Chavez-Abraham et al., 2011 [10]	Retrospective	425 (215 pump, 200 oral)	47.3 (pump) 49.7 (oral)	Pain infusion pump (lidocaine) vs. Oral narcotics	Using a continuous-infusion pain pump significantly reduced perceived pain and oral narcotic (Vicodin™) use postoperatively in patients undergoing augmentation mammaplasty or abdominoplasty
8	Singla et al., 2019 [26]	RCT	401 (81 placebo, 77 0.1 mg oliceridine, 80 0.35 mg oliceridine, 80 0.50 mg oliceridine, 83 1 mg morphine)	41.4 ± 10.2	Placebo vs. Oliceridine vs. Morphine	Oliceridine, at 0.35 and 0.5 mg doses, provided effective pain relief similar to morphine with fewer side effects in abdominoplasty patients, offering a viable alternative for acute pain management
9	Elsawy et al., 2021 [27]	RCT	51 (25 ESP, 26 TAP)	NIL	Erector spinae plane (ESP) block vs. Transverse abdominis plane (TAP) block	ESP block significantly lowered pain scores at 8 and 12 h, delayed the first analgesic need, and reduced 24 h pethidine consumption compared to the TAP block
10	Mansour et al., 2021 [28]	RCT	50 (25 L, 25 LK)	25–50	TAP block + levobupivacaine (L) vs. TAP block + levobupivacaine + ketamine (LK)	Adding 0.5 mg/kg ketamine to levobupivacaine for TAP blocks significantly reduced postoperative pain and morphine needs, and delayed the first request for rescue analgesia, improving analgesic effectiveness over levobupivacaine alone
11	Salama 2018 [29]	RCT	90 (30 TAP + levobupivacaine, 30 infusions + levobupivacaine, 30 saline)	41.6 ± 10.5 (TAP) 40.3 ± 11.7 (infusion) 42.1 ± 9.8 (saline) /25–50	TAP + levobupivacaine vs. Infusion + levobupivacaine vs. Saline	Continuous bilateral ultrasound-guided TAP block and local anesthetic wound infusion significantly reduced morphine use and hastened first morphine dose and ambulation times in the first 48 h post-surgery compared to placebo, with no significant difference between the two techniques
12	Alotaibi et al., 2020 [30]	Retrospective	60 (30 TAP, 30 no TAP)	NIL	TAP vs. No TAP	Patients receiving an ultrasound-guided TAP block post-lipoabdominoplasty needed significantly fewer postoperative opioids, had a longer time before their first analgesic request, had lower pain scores during mobilization, and experienced fewer instances of nausea and vomiting compared to the control group
13	Farahat et al., 2023 [31]	RCT	69 (23 standard analgesia, 23 TAP block, 23 ESP block)	25–65	Standard analgesia vs. TAP block vs. Erector spinae plane (ESP)	ESP-B and TAP-B blocks had similar efficacies in managing postoperative pain, reducing heart rate spikes, cortisol levels, and the need for rescue analgesia with similar outcomes, despite ESP-B’s longer application time
14	Kakagia et al., 2007 [32]	RCT	46	(NS) 36.80 ± 4.33, (R) 37.20 ± 6.84, (L) 40.27 ± 7.83	Normal saline (NS) vs. Ropivacaine (R) vs. Levobupivacaine (L)	In fleur-de-lys abdominoplasty, both ropivacaine and levobupivacaine reduced postoperative pain, with levobupivacaine showing superior effectiveness against pain intensity and duration
15	Sun et al., 2008 [33]	RCT	112 (39 peri-op, 37 post-op, 36 control)	42 ± 12 (control) 42 ± 14 (post-op) 43 ± 14 (peri-op)	Celcoxib Placebo Celcoxib vs. Placebo Celcoxib Celcoxib vs. Placebo Placebo Placebo	Celecoxib reduced postoperative pain, opioid need, and recovery time in plastic surgery, enhancing satisfaction without added preoperative benefit
16	Michaels et al., 2009 [34]	Retrospective	68 (39 Grp 1, 29 Grp 2)	Grp 1: 44 Grp 2: 43	General endotracheal analgesia (Grp 1) vs. Rib block (Grp 2)	Rib blocks before surgery significantly reduced recovery time, pain, postoperative narcotics, and nausea, enhancing patient comfort and making outpatient abdominoplasties more feasible without major complications
17	Gravante et al., 2011 [35]	Retrospective	51 (27 PB, 24 aesthet)	42	Post-bariatric (PB) vs. Aesthetic abdominoplasty with frank liposuction Both groups received TAP block	Revealed no complications from anesthesia or TAP block technique in abdominoplasty patients, with those undergoing post-bariatric surgery requiring more analgesia, potentially due to larger tissue resection
18	Meouchy et al., 2021 [36]	RCT	40 (20 ropivacaine, 20 normal saline)	Ropivacaine: 43.1 Saline: 45.6	Ropivacaine vs. Normal saline Both groups received a bilateral quadratus lumborum block	Quadratus lumborum block decreased postoperative pain, lowered opioid consumption, and enhanced recovery quality compared to controls
19	Sforza et al., 2024 [37]	RCT	30 (15 SPECS block, 15 normal saline)	23–32	Serratus anterior + Pectoral nerve block (S-PECS) vs. Normal Saline	S-PECS block significantly reduced pain and decreased postoperative medication needs in breast augmentation surgery
20	Feng 2010 [3]	Prospective	97 (77 combination nerve blocks, 20 no blocks)	Combination: 45.7 ± 9 No blocks: 46.9 ± 9	Intercostal + ilioinguinal + iliohypogastric + pararectus blocks (combination nerve blocks) vs. No blocks	Combining intercostal, ilioinguinal, iliohypogastric, and pararectus blocks in abdominoplasty provided long-term pain relief, reduced recovery time, and enhanced return to daily activities compared to controls
21	Araco et al., 2010 [38]	Retrospective	75 (34 TAP-B, 41 no TAP-B)	TAP-B: 39 ± 8 No TAP-B: 42 ± 10	TAP-B vs. No TAP-B	TAP-Bs were safe and effective for reducing postoperative opioid needs in aesthetic abdominal surgery, with no complications observed, but noted block failure in patients with higher BMI or larger flap resection, requiring additional analgesia
22	Wu et al., 2019 [39]	RCT	180 (60 LAI, 60 TL, 60 RTR)	48.0 ± 11.4 (LAI) 47.6 ± 10.1 (TL) 48.6 ± 12.1 (TR) /18–65	Local anesthetic infiltrate (LAI) vs. Ultrasound-guided posterior TAP-B (TL) vs. Ultrasound-guided subcostal TAP-B combined with RS-B (TR)	No significant differences in postoperative outcomes among those receiving different pain management techniques, but the LAI group reported higher satisfaction within 48 h post-surgery
23	Metry et al., 2019 [40]	RCT	200 (100 general anesthesia, 100 spinal anesthesia)	39.1 ± 10.2 (GA) 36.6 ± 8.8 (SA) /25–55	General anesthesia (GA) vs. Spinal anesthesia	Spinal group required less nalbuphine postoperatively, despite recording higher overall VAS scores than the general anesthesia group
24	Türkoglu et al., 2022 [41]	RCT	47 (16 tramadol, 16 ibuprofen, 15 ibuprofen + tramadol)	42.75 ± 13.17 (tramadol) 40.13 ± 11.13 (ibuprofen) 38.13 ± 7.88 (tramadol + ibuprofen) /18–65	IV tramadol vs. IV ibuprofen vs. IV tramadol + ibuprofen	VAS values were significantly lower in Group 3 compared to both Group 1 and Group 2, while Group 3 also exhibited significantly lower total analgesic consumption compared to Group 1

**Table 3:** Risk of Bias assessment of RCTs.

Article	Randomization Process	Deviations from Intended Intervention	Missing Outcome Data	Measurement of Outcome	Selection of Reported Results	Overall Risk
Ali et al., 2020 [20]	**Low** Random allocation achieved via computer-generated number table. Method of concealment was not explicitly mentioned	**Some concerns** No explicit mention of how deviations from intended interventions were managed	**Low** 160/180 patients completed the study. However, not mentioned how missing data and dropouts were handled	**Low** Objective measures like the VAS and Ramsey scale were used	**No information** No explicit mention of any trial protocol being followed	**Low**
Beaton et al., 2023 [11]	**Low** Randomization achieved using an electronic system. However, concealment method was not explicitly mentioned	**Some concerns** No explicit mention of how deviations from intended interventions were managed	**Low** Detailed description of trial process and follow-up were stated. Missing data likely addressed during these procedures	**Low** Patient-completed numerical pain rating scale and prespecified multiple timepoints for data collection indicates a standardized and objective approach to outcome measurement	**Low** Study protocol was registered. Use of statistical testing reduced risk of type I errors	**Low**
Abo-Zeid et al., 2018 [12]	**Low** Randomization achieved using closed envelope method. Concealment method not explicitly mentioned	**No information** Insufficient information on the management of and adherence to the intervention protocols	**No information** No information about how missing data and dropouts were handled.	**Low** Post-op data were assessed by an independent anesthetist who was not informed about the technique	**No information** No mention of study protocol	**High**
Minkowitz et al., 2020 [6]	**Some concerns** Details of randomization and concealment not provided	**Some concerns** No explicit information on how compliance was managed	**Low** Study used multiple imputation to handle missing data	**Low** SPID calculation was used to quantify efficacy of analgesia	**Low** Study was registered and conducted in accordance with the Helsinki Declaration	**Low**
Varas et al., 2020 [23]	**Low** SealedEnvelopeTM software used for randomization. Nurse unrelated to the project was asked to handle randomization, ensuring blinding	**Some concerns** No explicit mention of how deviations from intended interventions were managed	**Some concerns** No information about how missing data and dropouts were handled.	**Low** One-way ANOVA, Kruskal–Wallis test, and Fisher’s exact test used to measure outcomes	**Low** Study was registered and conducted in accordance with the relevant protocol	**Low**
Silva Filho et al., 2021 [24]	**Low** Electronic draw and opaque letters used to randomize participants. No explicit mention of concealment method	**Some concerns** No explicit mention of how deviations from intended interventions were managed	**Some concerns** No information about how missing data and dropouts were handled.	**Low** Two-way ANOVA used to measure primary outcome Verbal pain scale used to measure secondary outcome	**Some concerns** No mention of study protocol or registration	**High**
Mentz et al., 2005 [17]	**Some concerns** No details about how the randomization or concealment process were conducted	**Some concerns** No explicit information on how compliance was managed	**Some concerns** No information about how missing data and dropouts were handled.	**Some concerns** While the study uses quantity of analgesic pills taken as a measure of pain control, it does not utilize direct observational scales (e.g., visual analogue scale) to evaluate pain control	**Some concerns** No mention of study protocol or registration	**High**
Singla et al., 2019 [26]	**Some concerns** No details about how the randomization or concealment process were conducted	**Some concerns** No explicit information on how compliance was managed	**Low** Missing NRS pain scores were imputed by linear interpolation or a model-based multiple imputation method	**Low** Quantitative metrics were used to measure all outcomes Sufficient detail provided	**Low** Trial protocol approved by centralized institutional board through Advarra	**Low**
Elsawy et al., 2021 [27]	**Some concerns** No details about how the randomization or concealment process were conducted	**Some concerns** No explicit information on how compliance was managed	**Some concerns** No mention about how missing data were handled	**Low** Visual analogue scale used to measure pain felt by participants	**Low** Trial approved by Ethics and Scientific Committee at Al-Azhar University’s Faculty of Medicine in Cairo	**High**
Mansour et al., 2021 [28]	**Low** Randomization conducted using a computer-generated list and a sealed envelope method. However, details of concealment are not given	**Some concerns** No explicit information on how compliance was managed	**Some concerns** No mention about how missing data were handled	**Low** The primary outcome, VAS, is a subjective measure. The study mentions that an anesthesiologist blinded to group allocation conducted the assessments, which suggests a low risk of detection bias	**Low** Approval of the Ethical Committee of the Faculty of Medicine, Tanta University Hospital, followed by registration in the Pan African Clinical Trials Registry	**Low**
Salama 2018 [29]	**Low** Randomization conducted using a computer-generated list and a sealed envelope method. However, details of concealment are not given	**Some concerns** No explicit mention of how deviations from intended interventions were managed	**Some concerns** No mention about how missing data were handled	**Low** Primary and secondary outcomes were objectively measured. Blinded assessors in the PACU and ward nurses collected the data	**Some concerns** No mention of study protocol or registration	**High**
Farahat et al., 2023 [31]	**Low** Computer-generated algorithm and sealed envelope method used to randomize. However, no details about concealment were given	**Some concerns** No explicit mention of how deviations from intended interventions were managed	**Some concerns** No explicit mention of how missing data were handled	**Low** The outcomes, including total opioid consumption, time to first request for analgesia, and pain assessments, were clearly defined and measured	**Low** The study protocol was approved by the IRB at the Mansoura Faculty of Medicine. Clinical trial was registered in the ClinicalTrials.gov identifier	**Low**
Kakagia et al., 2007 [32]	**Low** Random number generator used for allocation. No explicit details about concealment were given	**Some concerns** No explicit mention of how deviations from intended interventions were managed	**Some concerns** No explicit mention of how missing data were handled	**Low** Patients self-reported their pain using a visual analog scale (VAS) and that standard monitoring was used postoperatively	**Some concerns** No mention of study protocol or registration	**High**
Sun et al., 2008 [33]	**Low** Computer-generated number schedule for patient allocation. All assessors were also blinded.	**Some concerns** No explicit mention of how deviations from intended interventions were managed	**Some concerns** No explicit mention of how missing data were handled	**Low** Outcomes were measured using standardized scales (VRS for pain, a 9-item questionnaire on quality of recovery), and an independent trained interviewer blinded to the study medication collected the data	**Low** IRB approval was achieved	**Low**
Singla et al., 2018 [18]	**Low** Random number generator used for allocation. No explicit details about concealment were given	**Some concerns** No explicit mention of how deviations from intended interventions were managed	**Some concerns** No explicit mention of how missing data were handled	**Low** Pain intensity was assessed using a standardized 11-point numeric pain rating scale, and the performance of the study medication was evaluated using a patient global assessment	**Low** Trial was approved by the IRB	**Low**
Sforza et al., 2024 [37]	**Low** The article clearly states that it is a randomized controlled trial, detailing who was responsible for the list and how they were absent during the procedure in the methods section	**Some concerns** No explicit information on how compliance was managed	**Low** All patients attended the mandatory appointments, suggesting that there were no missing outcome data and indicating a low risk of attrition bias	**Low** The study used a generalized estimating equation (GEE) with a logistic link to evaluate the effect of our main exposure, PECS, on the main outcome of pain medicine request, which was coded as a binary outcome (yes/no)	**Low** The study was approved by the hospital’s medical advisory board and adhered to the Declaration of Helsinki	**Low**
Wu et al., 2019 [39]	**Low** The study describes a detailed randomization method using numbered opaque envelopes, suggesting a good attempt to prevent selection bias.	**Some concerns** No explicit information on how compliance was managed	**Some concerns** There is no information provided about how missing outcome data were handled, so we cannot determine if there is a high or low risk of bias in this domain.	**Low** A visual analogue scale (VAS) was used	**Low** The study received ethical approval from The First People’s Hospital of Hefei’s Ethics Committee (Approval No. 2015-11), was registered with the Chinese Clinical Trial Registry (ChiCTR-IOR-16009912), and adhered to the Declaration of Helsinki principles. All participants provided written informed consent.	**Low**
Bjelland et al., 2019 [19]	**Low** The study utilized concealed computerized block randomization with a 1:1 allocation ratio, performed by the hospital pharmacy using an algorithm programmed in R	**Some concerns** The study lacks explicit discussion on adherence monitoring or deviation management	**Some concerns** The study does not detail how it handled any missing data	**Low** Outcomes were measured using objective criteria such as morphine equivalent units and numerical rating scales for pain, with methods like algometry for pain tolerance assessment	**Low** Registration with clinical trial registries (EudraCT and clinicaltrials.gov) supports transparency	**Low**
Meouchy et al., 2021 [36]	**Low** The study used an internet-based randomization program to randomly allocate patients. They also used sealed envelopes which were opened by an independent nurse who was not involved in the randomization	**Some concerns** The study does not explicitly mention how it handled deviations in intervention	**Some concerns** The study does not detail how missing data were handled	**Low** The study used the numeric rating scale to score pain	**Low** Ethical approval was obtained from the Institutional Review Board of Hotel-Dieu de France Hospital	**Low**
Metry et al., 2019 [40]	**Low** The study utilized a computer-created table of arbitrary numbers for random assignment	**Some concerns** The study does not explicitly mention how deviations in interventions, if any, were handled	**Low** The summary does not indicate any missing outcome data, suggesting that outcomes were reported for all participants	**Low** The study appropriately utilized the visual analogue scale (VAS) to analyze pain	**Low** The study is registered in ClinicalTrials.com with an ID number, and received ethical approval from the Ain Shams University Hospital	**Low**
Türkoglu et al., 2022 [41]	**Low** The study utilized a computer-created table of random numbers for allocating patients to treatment groups	**Some concerns** The description does not provide sufficient detail to assess whether any deviations from the planned interventions occurred and how they were handled	**Some concerns** The study does not mention how missing data were addressed, making it difficult to assess the impact of any missing outcome data on the study’s validity	**Low** The study utilized standardized methods for measuring outcomes, such as VAS scores for pain, and monitored vital signs	**Low** The study received approval from the Bolu Abant İzzet Baysal University Clinical Studies Ethics Committee	**Low**

**Table 4:** Risk of Bias assessment of cohort studies.

Article	Confounding	Participant Selection	Classification of Interventions	Deviations from Intended Interventions	Missing Outcome Data	Measurement of Outcomes	Selection of Reported Result	Overall Risk
Edwards et al., 2015 [13]	**Moderate** Confounding exists due to variability in surgical procedures	**Moderate** Selection criteria not clearly described	**Low** Interventions clearly described	**Moderate** No explicit information on deviation from intended interventions or adherence to protocol being monitored	**Moderate** No explicit information on how missing data were handled	**Moderate** Measures multiple outcomes. However, lacks details on the reliability and validity of the measurement tools	**Moderate** Reports multiple outcomes with statistical tests applied. Does not provide information on whether adjustments were made for multiple comparisons, which increases potential for type I error	**Moderate**
Giordano et al., 2020 [21]	**Moderate** No information about how confounding variables such as age, BMI, and medical history were controlled during allocation	**Moderate** Unclear representativeness of the study sample of the larger population	**Low** Interventions clearly described	**Moderate** No explicit information on deviation from intended interventions or adherence to protocol being monitored	**Moderate** No explicit information on how missing data were handled	**Moderate** Measures multiple outcomes. However, lacks details on the reliability and validity of the measurement tools	**Moderate** Not clear if all relevant outcomes were reported, or if unreported analyses exist	**Moderate**
Gardner et al., 2019 [14]	**Low** Participants were randomized, however small sample size and other potential confounding variables pose a risk	**Moderate** Criteria are clear, however small sample size and specific inclusion criteria may limit generalizability of findings	**Low** Intervention clearly described	**Moderate** No explicit information on deviation from intended interventions or adherence to protocol being monitored	**Moderate** No explicit information on how missing data were handled. Can be significant given small sample size	**Moderate** Measures multiple outcomes. However, lacks details on the reliability and validity of the measurement tools	**Moderate** Statistical methods and significance levels specified.	**Moderate**
Fiala 2015 [15]	**Low** Controls for confounding with detailed inclusion/exclusion criteria	**Moderate** Criteria for controlled and treatment groups not explicitly compared	**Low** Intervention clearly described	**Low** Technique of intervention is well detailed and study reports adherence to intended intervention	**High** No explicit information on how missing data were handled	**Low** Outcome measures relatively straightforward and measurement likely reliable	**Low** Study was registered and conducted in accordance with the relevant protocol	**Moderate**
Shauly et al., 2022 [22]	**Moderate** Appears to control for confounding factors through detailed data collection. However, the impact of potential unmeasured confounders cannot be fully assessed in a retrospective study.	**High** Inclusion/exclusion criteria not explicitly mentioned.	**Low** Interventions clearly described	**Moderate** No explicit information on deviation from intended interventions or adherence to protocol being monitored	**High** No explicit information on how missing data were handled	**Low** Patient-reported pain scored reported using visual analog score (VAS). Measurement appears reliable	**Low** Study was registered and conducted in accordance with the relevant protocol	**High**
Morales Jr. et al., 2013 [16]	**Moderate** Exclusion of 54 participants introduces selection bias	**High** Exclusion of 54 participants due to loss during follow-up introduces selection bias	**Low** Interventions clearly described	**Low** Study described a consistent intervention technique and a well-detailed method of administration	**High** Impact of missing data from 54 excluded participants on results not adequately addressed	**Moderate** Outcomes recorded using a subjective scale. No details on validity and reliability of pain management tool used	**Low** Study was registered and conducted in accordance with the relevant protocol	**High**
Price et al., 2023 [7]	**Moderate** Study adjusts for participant demographics but does not provide details on additional potential confounders such as comorbidities	**Low** Clear selection criteria with no exclusions	**Low** Interventions clearly described	**Moderate** Experimental group not given option in choice of analgesia	**Moderate** No explicit information on how missing data were handled	**Moderate** Some outcomes are clearly defined. Does not estimate partial prescription use of intervention	**Low** Reports outcomes and utilizes statistical analysis to compare different aspects of pain management between two groups. Bonferroni correction used, and alpha value adjusted to decrease potential for type I error	**Moderate**
Bray Jr. et al., 2007 [25]	**Moderate** Study does not provide details on how confounding variables are controlled	**Low** Clear selection criteria	**Low** Interventions clearly described	**Moderate** No explicit information on whether participants in certain group adhered to assigned intervention	**Moderate** No explicit information on how missing data were handled	**Moderate** Pain scores and medication were used at regular intervals, providing clear assessment of postoperative pain management. Did not adjust for multiple testing in statistical analysis which increases potential for type I error	**Moderate** Did not adjust for multiple testing which may impact reliability of results	**Moderate**
Chavez-Abraham et al., 2011 [10]	**Moderate** Study does not provide details on how confounding variables are controlled	**Low** Clear selection criteria	**Moderate** Interventions clearly defined. Vague explanation on administration of lidocaine and its comparison	**Moderate** No explicit information on participant adherence to intervention	**Moderate** No explicit information on how missing data were handled	**Moderate** Lack of details on methods used for pain assessment	**Moderate** Reports outcomes related to pain level and narcotic use. Does not specify any statistical adjustments made for multiple comparisons which increases potential for type I error	**Moderate**
Alotaibi et al., 2020 [30]	**Moderate** Study groups are compared for multiple variables, but still confounding variables are not addressed	**Moderate** Well defined inclusion/exclusion criteria but participants not randomized	**Low** Interventions clearly described	**Moderate** No explicit information on participant adherence to intervention	**Moderate** No explicit information on how missing data were handled	**Moderate** Measures multiple outcomes. However, lacks details on the reliability and validity of the measurement tools.	**Moderate** Reports multiple outcomes with statistical tests applied. Does not provide information on whether adjustments were made for multiple comparisons which increases potential for type I error	**Moderate**
Michaels et al., 2009 [34]	**Moderate** Study groups are compared for multiple variables, but still confounding variables are not addressed	**Moderate** Well defined inclusion criteria but participants not randomized	**Low** Interventions clearly described	**Moderate** No explicit information on deviation from intended interventions or adherence to protocol being monitored	**Moderate** No explicit information on how missing data were handled	**Moderate** Measures multiple outcomes. However, it lacks details on the reliability and validity of the measurement tools.	**Moderate** Reports multiple outcomes with statistical tests applied. Does not provide information on whether adjustments were made for multiple comparisons which increases potential for type I error	**Moderate**
Gravante et al., 2011 [35]	**Moderate** Study groups are compared for multiple variables, but still confounding are variables not addressed	**Moderate** Inclusion criteria not clearly defined	**Low** Interventions clearly described	**Moderate** No explicit information on deviation from intended interventions or adherence to protocol being monitored	**Moderate** No explicit information on how missing data were handled	**Moderate** Measures multiple outcomes. However, it lacks details on the reliability and validity of the measurement tools.	**Moderate** Reports multiple outcomes with statistical tests applied. Does not provide information on whether adjustments were made for multiple comparisons	**Moderate**
Araco et al., 2010 [38]	**Moderate** The study does not account for other factors like preoperative health status or individual pain thresholds	**Moderate** Inclusion criteria only account for patients who underwent flank liposuction whereby the TAP technique was used	**Low** Interventions are well defined, with clear descriptions of the TAP technique and post-op care protocol	**Some concerns** The study does not go into detail on adherence to intended surgical and post-op protocols	**Some concerns** The study does not delineate how missing data were handled	**Low** The outcomes measured are objective (e.g., total bupivacaine injected, post-op morphine required)	**Low** Reports multiple outcomes with statistical tests applied	**Moderate**
Feng 2010 [3]	**Low** A substantial number of factors were considered (age, gender, BMI, date and type of procedure, types and composition of blocks, pain score)	**Moderate** Patients were divided based on the period in which they underwent surgery, which might correlate with changes in practice over time rather than the intervention itself	**Low** The interventions are well described and classified, providing clear differentiation between treatment and control groups	**Some concerns** There is no information on whether there were any deviations from the planned interventions or how they were managed	**Some concerns** The study does not mention how missing data, if any, were handled	**Moderate** Outcomes were measured using standardized tools such as the visual analogue scale for pain and opioid equivalence charts for narcotics. However, the reliance on recovery room records and patient questionnaires sent 6 weeks postoperatively may introduce recall bias or variability in how outcomes were reported	**Low** Reports multiple outcomes with statistical tests applied such as the t-test and Mann–Whitney U test	**Moderate**

## Data Availability

The authors confirm that the data supporting the findings of this study are available within the article.
